# Designing B_1_ ‐Selective Pulses by Frequency Modulating in a Second Rotating Frame

**DOI:** 10.1002/mrm.70259

**Published:** 2026-01-18

**Authors:** Saurin Kantesaria, Efraín Torres, Mazin M. Mustafa, Djaudat Idiyatullin, Sara Ponticorvo, Gregor Adriany, Shalom Michaeli, Michael Garwood

**Affiliations:** ^1^ Center for Magnetic Resonance Research University of Minnesota Minneapolis Minnesota USA

**Keywords:** adiabatic pulses, B_1_ gradients, low‐cost MRI, RF pulse design, RF‐encoded MRI, slice selection

## Abstract

**Purpose:**

To create a frequency‐modulated pulse for slice selection of arbitrary, uniform flip angle even when *B*_0_ and *B*_1_ are inhomogeneous, without utilizing B_0_ gradients

**Theory and Methods:**

An amplitude‐modulated hyperbolic secant pulse (AM_HS1_) was derived from Hoult's B_1_‐selective imaging method which utilizes one component, *B*_1y_, as a gradient and another, *B*_1x_, for selective excitation. The frequency sweep in AM_HS1_ was produced by a time‐dependent amplitude‐modulation of *B*_1x_, defining the band of nutation frequencies to select along the *B*_1y_ gradient. Resilience of slice selection to *B*_0_ and *B*_1_ inhomogeneities was investigated by simulations. Slice‐ and slab‐selective imaging were demonstrated experimentally in phantoms and rat brain in vivo using surface coils.

**Results:**

Simulations of AM_HS1_ demonstrated slice inversion despite *B*_0_ and *B*_1_ inhomogeneities. When operating sub‐adiabatically to produce excitation flip angles < 180° with a single coil, the flip angle across the slice varied because both *B*_1x_ and *B*_1y_ gradients were present. This problem was corrected by scaling *B*_1x_(*t*) by the normalized frequency‐sweep and phase‐modulation functions. Slice selection using only a *B*_1_ gradient was demonstrated on phantoms using a pair of AM_HS1_ pulses transmitted with a surface coil. By using a low‐flip angle AM_HS1_ with *B*_1y_ refocusing lobes, slice‐ and slab‐selective excitation was realized in 3D gradient‐echo imaging of rat brain in vivo at 9.4 T.

**Conclusion:**

By implementing frequency modulation in a second rotating frame, B_1_‐selective excitation and inversion are feasible, even when *B*_0_ and *B*_1_ are nonuniform.

## 1 Introduction

Typical clinical MRI scanners are expensive to purchase and operate. The most expensive component is the magnet producing the static field ($B_{0}$). The second most costly component is typically the $B_{0}$‐gradient system [1]. In addition, $B_{0}$ gradient systems require expensive gradient amplifiers with infrastructure costs from power and cooling. Gradient coils also occupy precious bore space, and when switched on and off quickly, produce Lorentz forces generating loud noise which increases patient anxiety [2].

Radiofrequency (RF) gradients offer an exciting alternative to $B_{0}$ gradients for spatial encoding. They save bore space and scan quietly. Furthermore, RF gradients can be switched faster (∼100‐fold) than $B_{0}$ gradients, opening new possibilities for pulse sequence design. Minimal to no cooling is needed, reducing long term costs as well.

The first RF‐gradient‐encoding method was Hoult's rotating frame zeugmatography [3]. While it established a foundation for other RF‐encoding methods, it can have high specific absorption rate (SAR) and therefore is not clinically practical. Other methods such as B_1_‐selective pulses (Hoult's rotating frame pulses [4, 5], *Shinnar‐Le‐Roux*‐optimized (SLR) [6], SLR‐optimized with Bloch‐Siegert shift [7, 8, 9], etc.), Transmit Array Spatial Encoding (TRASE) [10], and Frequency‐modulated Rabi Encoded Echoes (FREE) [11] have since built off Hoult's work to move towards lower cost MRI without $B_{0}$ gradients. Of these, only three, rotating frame zeugmatography [4, 5], TRASE [10], and B_1_‐selective pulses [4, 5, 6, 7, 8, 9], can perform slice selection, a key method needed for nearly all mainstay sequences of clinical MRI. FREE and TRASE acquire one data point per acquisition and therefore require multi‐shot or multi‐echo acquisitions for localization. As such they are not ideal options for slice selection. B_1_‐selective pulses are then the most practical, but are limited by high SAR and sensitivity to resonance offset, $\Omega =\omega_{0}-\omega_{c}\neq$ 0, where $\omega_{c}$ is the carrier frequency and $\omega_{0}$ is the Larmor frequency [4, 5, 6, 7, 8, 9].

Adiabatic pulses, such as hyperbolic secant (HS) pulses, are robust to resonance offsets [12] as they produce a frequency sweep that sequentially tips spins within any specified bandwidth. In this work, we show how adiabatic pulses like the original HS pulse (HS1) can be implemented in the rotating frame to enable slice and slab selection using only an RF gradient.

Hoult's method [4, 5] uses two RF fields, $B_{1x}$ and $B_{1y}$, which respectively serve the same purpose as $B_{1}$‐ and $B_{0}$‐gradient fields in conventional MRI. $B_{1x}$ is applied uniformly over the object, whereas $B_{1y}\left(r\right),$ is applied as a gradient in direction r to create a continuum of nutation frequencies (aka Rabi frequencies). A band of these Rabi frequencies can be excited selectively by $B_{1x}$. Originally, $B_{1x}$ was applied at a constant frequency; however, as we show in this work, if a frequency‐swept adiabatic pulse like HS1 [13, 14] is used instead, then resilience to $B_{1}$ and $B_{0}$ inhomogeneity can be achieved.

Here, we introduce a method to perform slice and slab selection without $B_{0}$ gradients by applying Hoult's framework to an amplitude‐modulated (AM) adiabatic HS1 pulse (AM_HS1_). Slice inversion and its resilience to $B_{0}$ and $B_{1}$ inhomogeneity is demonstrated by simulations and experiments in phantoms and in rats in vivo at 9.4 T using surface coils. Further, we demonstrate that a correction can be applied to AM_HS1_ to produce a boxcar‐shaped slice profile of arbitrary flip angle.

## 2 Theory

### 2.1 Two‐Coil B_1_ ‐Selective Slice Selection

In Hoult's method, two separate $B_{1}$ fields are applied, $B_{1x}$ and $B_{1y}$[4, 5]. Consider a 1D distribution of spins in a spatially‐varying RF field applied at the Larmor frequency,

(1) $$\omega_{0}=\gamma B_{0},$$

where γ is the proton gyromagnetic ratio. In the first rotating frame (*x*′,*y*′,*z*′), the spatially‐varying component, $B_{1y}$, is arbitrarily oriented along *y*′. The Rabi frequency of a magnetization vector M then varies with position (*r*) in the RF gradient

(2) $$\begin{array}{c} \omega_{1y}\left(r\right)=\gamma B_{1y}\left(r\right). \end{array}$$

Simultaneously, a separate orthogonal, homogeneous RF field along *x*′, $B_{1x}$, is applied at the Rabi frequency at the slice center ($r_{\text{center}}$). With this, a band of nutating M vectors are tipped out of their nutation around $B_{1y}$, and thus, are selectively excited. Typically, the carrier frequency of $B_{1x}$ is set to $\omega_{0}$, while the amplitude of $B_{1x}$ is sinusoidally modulated at frequency $\omega_{1y}\left(r_{\text{center}}\right)$, resulting in a net frequency $\omega_{0}\pm \omega_{1y}\left(r_{\text{center}}\right)$.

**FIGURE 1:**
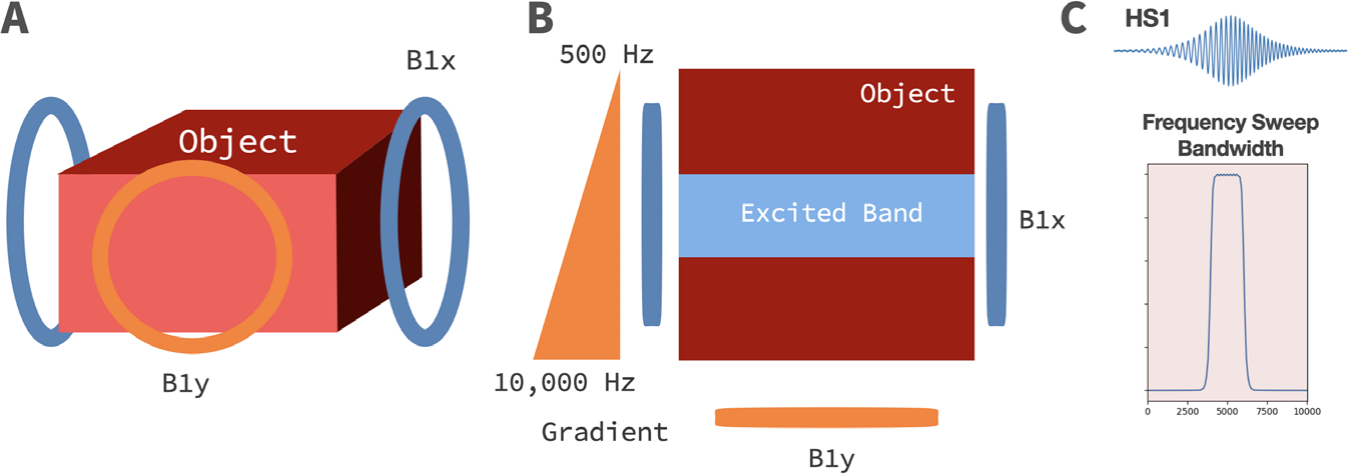
$B_{1}$‐selective rotating frame imaging with an adiabatic hyperbolic secant (HS1) pulse, AM_HS1_. (A) Object positioned with $B_{1x}$ and $B_{1y}$ coils. (B) Top‐down view of object denoting $B_{1x}$ as a homogeneous field, whereas $B_{1y}$ is a gradient field producing Rabi frequencies ($\frac{{\gamma B}_{1y}}{2\pi }$ = 500–10 000 Hz). $B_{1x}$ excites a band of spins along the $B_{1y}$ gradient when applied as an HS1 pulse. (C) $B_{1x}$ is applied as an HS1 pulse, here with a frequency sweep from ∼3000 to 6000 Hz, matching the excited band.

In the following method, the frequency of $B_{1x}$ is varied continuously, to create a frequency‐swept pulse in the second rotating frame. This pulse sweeps through Rabi frequencies of interest to sequentially tip spins similar to how HS1 tips a band of Larmor frequencies in the first rotating frame. We refer to this new B_1_‐selective pulse as AM_HS1_. Figure 1 illustrates this with two RF coils, although a single coil implementation is also described further below.

#### 2.1.1 Derivation of the Effective Field in the Second Rotating Frame

The equation of motion in the laboratory frame (neglecting relaxation) is

(3) $$\begin{array}{c} \frac{dM}{\mathrm{dt}}=\gamma M\times B. \end{array}$$

A transformation to a frame ($\left[x^{\prime },y^{\prime },z^{\prime }\right]$) rotating at frequency ω about *z* is then

(4) $$\begin{array}{c} \frac{\partial M^{\prime }}{\partial t}=\frac{dM}{\mathrm{dt}}-M\times \omega =M\times \left(\gamma B_{0}-\omega \right), \end{array}$$

where

(5) $$\begin{array}{c} B_{\mathrm{eff}}^{\prime }=\left(B_{0}-\frac{\omega }{\gamma }\right)\hat{z^{\prime }} \end{array}$$

is the effective field in this “first” rotating frame. $B_{\mathrm{eff}}^{\prime }$ is the familiar off‐resonance field. A second rotation about the $y^{\prime }$ axis at frequency $\omega_{1}^{\prime }$ results in a doubly rotating frame ($\left[x^{\prime \prime },y^{\prime \prime },z^{\prime \prime }\right]$),

(6) $$\begin{array}{c} \frac{\partial M^{\prime \prime }}{\partial t}=\frac{dM^{\prime }}{\mathrm{dt}}-M^{\prime }\times \omega_{1}^{\prime }=M^{\prime }\times \left(\gamma B_{\mathrm{eff}}^{\prime }-\omega_{1}^{\prime }\right), \end{array}$$

where the effective field is

(7) $$\begin{array}{c} B_{\mathrm{eff}}^{\prime \prime }\left(t\right)={B_{\mathrm{eff}}^{\prime }-\frac{\omega_{1}^{\prime }}{\gamma \;}=B}_{\mathrm{eff}}^{\prime }\left(\cos\left(\omega_{1}^{\prime }t\right)\hat{z^{\prime \prime }}-\sin\left(\omega_{1}^{\prime }t\right)\hat{x^{\prime \prime }}\right)-\left(\frac{\omega_{1}^{\prime }}{\gamma }\right)\hat{y^{\prime \prime }}. \end{array}$$

For more straightforward pulse sequence design and simulation, this equation can be expressed in terms of unit vectors in the first rotating frame as.

(8) $$\begin{array}{c} B_{\mathrm{eff}}^{\prime \prime }\left(t\right)=B_{\mathrm{eff}}^{\prime }\hat{z^{\prime }}-\left(\frac{\omega_{1}^{\prime }}{\gamma }\right)\hat{y^{\prime }}, \end{array}$$

given $\hat{y^{\prime }}=\hat{y^{\prime \prime }}.$ This simplification can be made as $\hat{z^{\prime }}$, when observed in the second rotating frame, rotates in the *x*″z″‐plane at ${-\omega }_{1}^{\prime }$. Note that a finite resonance offset leads to a potentially undesirable circularly‐polarized interference from $B_{\mathrm{eff}}^{\prime }\hat{z^{\prime }}$.

#### 2.1.2 Creating a Rotating Effective Field in the Second Rotating Frame

To create a frequency‐swept effective field in the second frame, two orthogonal time‐dependent components of the field are needed. These are $B_{1x}^{\prime \prime }\left(t\right)\hat{x^{\prime \prime }}$ and a $\hat{y^{\prime \prime }}$ vector representing a new resonance offset. This offset is the difference between the amplitude modulation frequency of $B_{1x}^{\prime \prime }\left(t\right)$ and the frequency of frame rotation, $\omega_{1}^{\prime }$. For simplicity, assume $B_{\mathrm{eff}}^{\prime }=0$. Similar to how $B_{\mathrm{eff}}\left(t\right)$ sweeps in the frequency‐modulated frame [12], the effective field in the second frame is

(9) $$\begin{array}{c} B_{\mathrm{eff}}^{\prime \prime }\left(t\right)=B_{1x}^{\prime \prime }\left(t\right)\hat{x^{\prime \prime }}-\frac{\omega_{1}^{\prime }\left(t\right)}{\gamma }\hat{y^{\prime \prime }}, \end{array}$$

where $\omega_{1}^{\prime }\left(t\right)$ is now variable in time to create this sweep.

#### 2.1.3 Hyperbolic Secant Pulses in the Second Rotating Frame

With a B_1_‐selective HS pulse, the amplitude of $B_{1x}^{\prime \prime }\left(t\right)\hat{x^{\prime \prime }}$ is a sech modulation and the offset field $B_{1y}^{\prime \prime }\left(t\right)\hat{y^{\prime \prime }}$ is a tanh modulation. This is analogous to the frequency modulation that creates the fictitious field along $z^{\prime }$ for a typical HS1 pulse in the frequency‐modulated frame. For AM_HS1_,

(10) $$\begin{array}{c} B_{1x}^{\prime \prime }\left(t\right)=B_{1x}^{\max}F_{1}\left(t\right) \end{array}$$

where

(11) $$\begin{array}{c} F_{1}\left(t\right)=\mathrm{sech}\left(\frac{2\beta }{T_{p}}\left(t-\frac{T_{p}}{2}\right)\right) \end{array}$$

and

(12) $$\begin{array}{c} B_{1y}^{\prime \prime }\left(t\right)=\frac{2\pi A}{\gamma }\tanh\left(\frac{2\beta }{T_{p}}\left(t-\frac{T_{p}}{2}\right)\right)\; \end{array}$$

where $B_{1x}^{\max}$ is the maximum $B_{1x}^{\prime \prime }\left(t\right)$, $F_{1}\left(t\right)$ is a normalized shape function, t is time, $T_{p}$ is pulse duration, 2*A* is the frequency sweep bandwidth in Hz, and truncation factor *β* = arcsech(0.01). Assuming $B_{\mathrm{eff}}^{\prime }=0$, this second frame HS1 can adiabatically rotate a magnetization vector from $y^{\prime }$ to $-y^{\prime }$ (noting again, $y^{\prime }=y^{\prime \prime }$).

Implementation using conventional MRI spectrometers requires the pulse driving function

(13) $$\begin{array}{c} B_{1\mathrm{xz}}^{\prime }\left(t\right)=B_{1x}^{\max}F_{1}\left(t\right)\left(\cos\left(\phi_{\mathrm{HS}1}\left(t\right)\right)\hat{x^{\prime }}+\sin\left(\phi_{\mathrm{HS}1}\left(t\right)\right)\hat{z^{\prime }}\right), \end{array}$$

where

(14) $$\begin{array}{c} \phi_{\mathrm{HS}1}\left(t\right)=\frac{\pi R}{2\beta }\ln\left(\frac{B_{1x}^{\max}}{B_{1x}^{\prime \prime }\left(t\right)}\right) \end{array}$$

is the time‐dependent angle that produces a time‐dependent field along $y^{\prime \prime }$. R is the unitless time‐bandwidth product. $B_{1y}^{\prime }\left(t\right)$ will later become a gradient to allow for spatial selectivity.

According to Equation (13), $B_{1\mathrm{xz}}^{\prime }\left(t\right)$ is circularly polarized in the x′z′ plane. To create the $z^{\prime }$ component of $B_{1\mathrm{xz}}^{\prime }\left(t\right)$, a z‐coil capable of sweeping between ±*A* with amplitude ${\gamma B}_{1x}^{\max}/2\pi$ ≥ *A* is needed. Because a z‐coil is not typically available, an alternative implementation uses the linearly‐polarized driving function,

(15) $$\begin{array}{c} B_{1x}^{\prime }\left(t\right)=B_{1x}^{\max}F_{1}\left(t\right)\left(\cos\left(\phi_{\mathrm{HS}1}\left(t\right)\right)\hat{x^{\prime }}\right), \end{array}$$

because $B_{1x}^{\prime }$ can be decomposed into two counterrotating fields,

(16) $$\begin{array}{cc} B_{1x}^{\prime }\left(t\right) & =\frac{B_{1x}^{\max}F_{1}\left(t\right)}{2}((\cos\left(\phi_{\mathrm{HS}1}\left(t\right)\right)\hat{x^{\prime }}+\sin\left(\phi_{\mathrm{HS}1}\left(t\right)\right)\hat{z^{\prime }})\\ & \;+\left(\cos\left(\phi_{\mathrm{HS}1}\left(t\right)\right)\hat{x^{\prime }}-\sin\left(\phi_{\mathrm{HS}1}\left(t\right)\right)\hat{z^{\prime }}\right)). \end{array}$$

The oppositely‐rotating component is neglected based on the rotating wave approximation [15], giving

(17) $$\begin{array}{c} B_{1x}^{\prime }\left(t\right)=\frac{B_{1x}^{\max}F_{1}\left(t\right)}{2}\left(\cos\left(\phi_{\mathrm{HS}1}\left(t\right)\right)\hat{x^{\prime }}+\sin\left(\phi_{\mathrm{HS}1}\left(t\right)\right)\hat{z^{\prime }}\right), \end{array}$$

and accordingly, $B_{1\mathrm{xz}}^{\prime }\left(t\right)=2B_{1x}^{\prime }\left(t\right)$. Of note, two amplitude‐modulations occur simultaneously in AM_HS1_. The first is the sech envelope from $F_{1}\left(t\right)$, while the second is due to the phase modulation $\phi_{\mathrm{HS}1}\left(t\right)$. This second modulation is fed into the amplitude‐modulation function for $B_{1x}^{\prime }\left(t\right)$ which forms the frequency sweep. This amplitude modulation is equivalent to two counterrotating fields that sweep at a varying rate in time (Figure S1).

#### 2.1.4 AM_HS1_ Pulse With Two RF Coils in the First Rotating Frame

Now consider two RF coils producing $B_{1x}^{\prime }\left(t\right)$ and $B_{1y}^{\prime }\left(t,r\right)$ in the first rotating frame to create an AM_HS1_ pulse. $B_{1x}^{\prime }\left(t\right)$ corresponds to a homogenous coil over the object and $B_{1y}^{\prime }\left(t,r\right)$ corresponds to a coil producing a $B_{1}$ gradient. $B_{1y}^{\prime }\left(t,r\right)$ will be a square pulse simplifying to $B_{1y}^{\prime }\left(r\right)$. Based on the above, $B_{1x}^{\prime }\left(t\right)$ is simply Equation (15). The frequency sweep FM(t) is simply the derivative of the phase modulation of Equation (14),

(18) $$\begin{array}{c} \mathrm{FM}\left(t\right)=\frac{d\phi_{\mathrm{HS}1}}{\mathrm{dt}}. \end{array}$$

The FM(t) function plays a critical role in ensuring acceptable performance of any FM pulse; however, implementation on MRI scanners typically requires the modulated phases of the pulse (Equation 14). To shift a selected slice along the *y*‐axis, the amplitude modulation of $B_{1x}^{\prime }\left(t\right)$ that is created by the phase modulation (*ϕ*_HS1_(*t*)) must be offset. For the two‐coil case, the total phase function modulating $B_{1x}^{\prime }\left(t\right)$ is then

(19) $$\begin{array}{c} \phi_{\mathrm{tot}}\left(t\right)=\phi_{\mathrm{HS}1}\left(t\right)+\phi_{\mathrm{off}}\left(t\right), \end{array}$$

where

(20) $$\begin{array}{c} \phi_{\mathrm{off}}\left(t\right)=2\pi f_{\mathrm{off}}t \end{array}$$

is a linear phase ramp calculated from the frequency offset $f_{\mathrm{off}}$ needed to position the slice in Rabi frequency space. Accordingly, $B_{1x}^{\prime }\left(t\right)$ is doubly amplitude‐modulated as.

(21) $$\begin{array}{c} B_{1x}^{\prime }\left(t\right)\;=B_{1x}^{\max}F_{1}\left(t\right)\cos\left(\phi_{\mathrm{tot}}\left(t\right)\right). \end{array}$$

**FIGURE 2:**
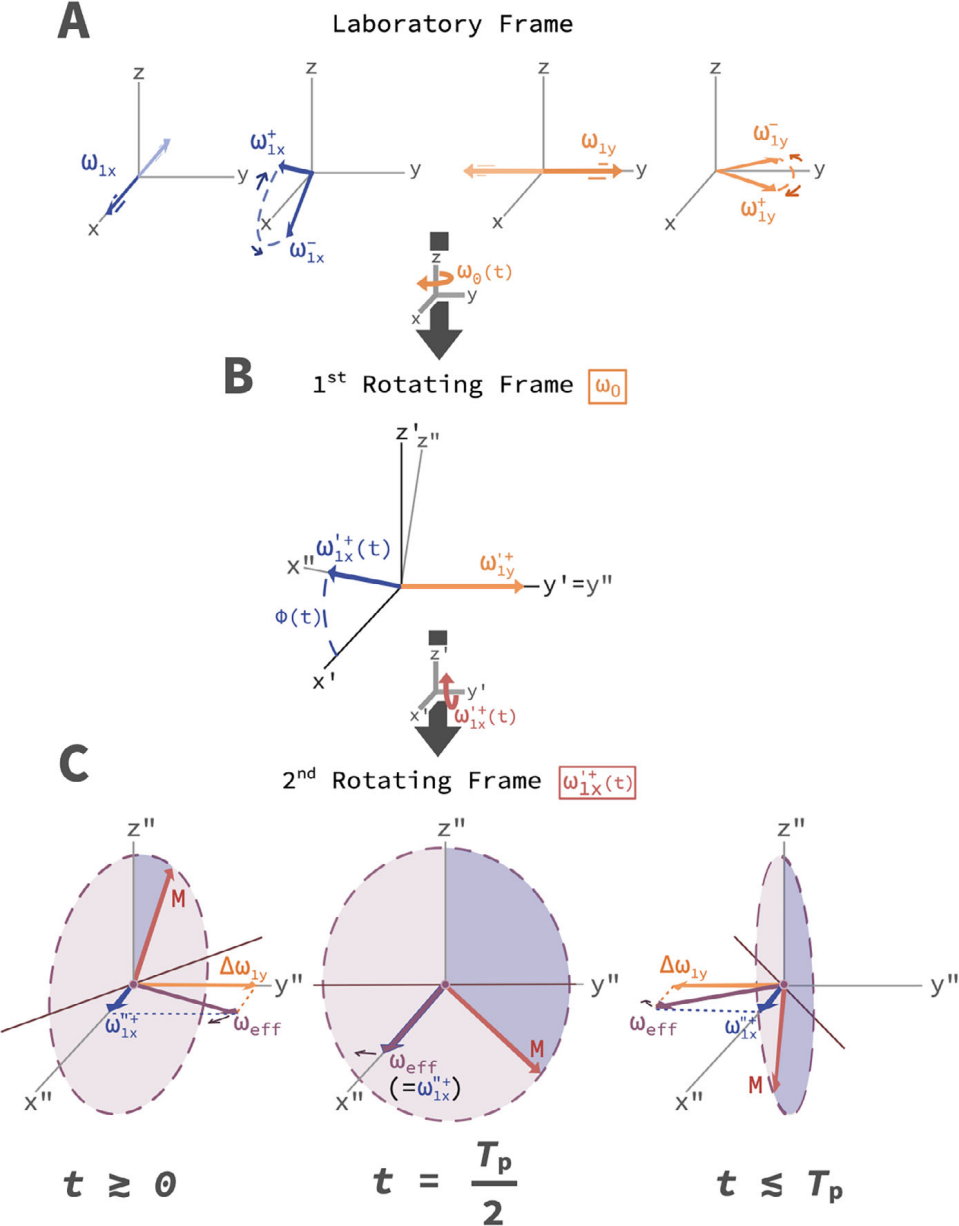
$B_{1}$‐selective rotating frame vector diagrams. (A) Lab frame: $\omega_{1y}\left(t\right)$ and $\omega_{1x}\left(t\right)$ are decomposed to counterrotating fields with amplitudes $\omega_{1x}^{\pm }$ and $\omega_{1y}^{\pm }$ on the *xz*‐plane (left) and *xy*‐plane (right). (B) First rotating frame: $\omega_{1y}^{\prime +}$ is along $y^{\prime }$ and $\omega_{1x}^{\prime +}\left(t\right)$ rotates about $y^{\prime }$ in the *x*′*z*′‐plane. (C) Second frame rotating at frequency $\omega_{1x}^{\prime +}\left(t\right)$ about $y^{\prime }$: $\omega_{1x}^{\prime \prime +}\left(t\right)$ becomes stationary along x″ and an effective field along y” appears, $\Delta \omega_{1y}\left(t\right).$ When $\omega_{1x}^{\prime +}\left(t\right)\neq \omega_{1y}^{\prime },$ $\Delta \omega_{1y}\left(t\right)=\left(\omega_{1x}^{\prime +}\left(t\right)-\omega_{1y}^{\prime }\right)\hat{y^{\prime \prime }}$. Given a symmetric frequency sweep, ${\Delta \omega }_{1y}\left(t\right)$ starts off large along +*y*” (left), then decreases to 0 (middle), and grows back to the same magnitude along −*y*” (right). This produces a net effective field $\omega_{\mathrm{eff}}\left(t\right)=\omega_{1x}^{\prime \prime +}\left(t\right)+{\Delta \omega }_{1y}\left(t\right)$ which **M** nutates around as it sweeps from right to left during the pulse.

The first amplitude modulation is through the HS1 shape function, scaled to produce $B_{1x}^{\max}F_{1}\left(t\right)$, whereas the second amplitude modulation is from the phase modulation $\phi_{\mathrm{tot}}\left(t\right)$ used to create a frequency sweep with a slice offset. A cosine is used to initially align the first and second frames in Figure 2.

### 2.2 B_1_ ‐Selective Slice With One RF Coil

While the theory for two RF coils is more straightforward, a single RF coil is preferred to reduce hardware requirements. For this, the $B_{1x}-\text{coil}$ is omitted and both components are applied using a single coil whose amplitude $B_{G}$ and phase $\phi_{\mathrm{RF}}$ are calculated from the two‐coil fields, $\;B_{1x}$ and $B_{1y}$. The gradient of the single coil is expressed as

(22) $$\begin{array}{c} B_{G}^{\prime }\left(t,r\right)=B_{G}\left(r\right)e^{i\phi_{\mathrm{RF}}\left(t\right)}, \end{array}$$

where the position‐dependent scalar $B_{G}$ describes the RF amplitude across the RF gradient of a single coil as

(23) $$\begin{array}{c} B_{G}\left(r\right)≔\sqrt{{\left(B_{1x}^{\max}\left(r\right)\right)}^{2}+{\left(B_{1y}^{\prime }\left(r\right)\right)}^{2}}, \end{array}$$

where $B_{1x}^{\max}\left(r\right)$ is the peak value of $B_{1x}^{\prime }\left(t,r\right)$ in time, and $B_{1y}^{\prime }\left(r\right)$ is a time‐independent orthogonal component, both defined according to the two‐coil equations above. The phase for the one‐coil pulse $\phi_{\mathrm{RF}}\left(t\right)$ (again with $B_{1y}^{\prime }$ and $B_{1x}^{\prime }$ from the two‐coil case) is

(24) $$\begin{array}{c} \phi_{\mathrm{RF}}\left(t\right)=\arctan\left(\frac{B_{1y}^{\prime }\left(r_{\text{center}}\right)}{B_{1x}^{\prime }\left(t,r_{\text{center}}\right)}\right). \end{array}$$

For convenience, hereafter we use vectors having units of frequency,

(25) $$\begin{array}{c} \omega_{1x}^{\prime }\left(t\right)=\gamma B_{1x}^{\prime }\left(t\right)\hat{x^{\prime }}, \end{array}$$

(26) $$\begin{array}{c} \omega_{1y}^{\prime }\left(r\right)=\gamma B_{1y}^{\prime }\left(r\right)\hat{y^{\prime }}. \end{array}$$

Likewise, the RF amplitude scalar is

(27) $$\begin{array}{c} \omega_{G}^{\prime }\left(r\right)=\gamma B_{G}\left(r\right). \end{array}$$

Figure 2 illustrates how the field components form in different frames. In the doubly‐rotating second frame that follows one of two counterrotating $\omega_{1x}^{\prime }\left(t\right)$ components, $\omega_{1x}^{\prime +}\left(t\right)$ (or $\omega_{1x}^{\prime -}\left(t\right)$), the component along $y^{\prime \prime }$, $\omega_{1y}^{\prime }$, disappears when $\omega_{1x}^{\prime +}\left(t\right)$ is the Rabi frequency. Likewise, a nutational off‐resonance field, $\;\Delta \omega_{1y}\left(t\right)=\left(\omega_{1x}^{\prime +}\left(t\right)-\omega_{1y}^{\prime }\right)\hat{y^{\prime \prime }}$, exists along $y^{\prime \prime }$ when $\omega_{1x}^{\prime +}\left(t\right)\neq \omega_{1y}^{\prime }$. Due to the frequency sweep, an effective field vector, $\omega_{\mathrm{eff}}\left(t\right)=\omega_{1x}^{\prime +}\left(t\right)+{\Delta \omega }_{1y}\left(t\right)$, traces from $-y^{\prime \prime }$ to $+y^{\prime \prime }$ (Figure 2).

### 2.3 Sub‐Adiabatic Excitation With One Coil

A fully adiabatic AM_HS1_ pulse will invert spins completely within its bandwidth for both two‐ and one‐coil setups. A sub‐adiabatic pulse with a single coil produces a slice with varying flip angle because $B_{1x}^{\prime }$varies spatially. In this scenario, the pulse will leave some magnetization along the *x*′‐axis. This dispersive component can be subtracted by cycling the initial phase between 0° and 180°. However, an undesirable effect remains due to dependence of $B_{1x}^{\max}$ on spatial position. To correct for this, the pulse amplitude can be scaled by a normalized version of the frequency sweep FM(t),

(28) $$\begin{array}{c} FM_{\mathrm{norm}}\left(t\right)=\frac{-\mathrm{FM}\left(t\right)}{\omega_{1y}^{\prime }\left(r_{\text{center}}\right)+\frac{\pi R}{T_{p}}}, \end{array}$$

and the phase‐modulation $\phi_{\mathrm{HS}1}\left(t\right),$

(29) $$\begin{array}{c} \phi_{\mathrm{norm}}\left(t\right)=\frac{\phi_{\mathrm{HS}1}\left(t\right)-\min\;\phi_{\mathrm{HS}1}\left(t\right)}{\max\phi_{\mathrm{HS}1}\left(t\right)-\min\phi_{\mathrm{HS}1}\left(t\right)}, \end{array}$$

as

(30) $$\begin{array}{c} F_{1_{\mathrm{cor}}}\left(t\right)=F_{1}\left(t\right)FM_{\mathrm{norm}}\left(t\right)\phi_{\mathrm{norm}}\left(t\right), \end{array}$$

where $FM_{\mathrm{norm}}\left(t\right)$ is the normalized frequency sweep, $\phi_{\mathrm{norm}}\left(t\right)$ is the normalized phase‐modulation, and $F_{1_{\mathrm{cor}}}\left(t\right)$ is the corrected shape function. To keep the amplitude constant, $F_{1_{\mathrm{cor}}}\left(t\right)$ is divided by its maximum. $FM_{\mathrm{norm}}\left(t\right)$ reverses and scales the frequency sweep based on the maximum frequency attained within its bandwidth to provide an initial scaling of the shape function based on position within the $B_{1x}^{\prime }$ gradient. For large bandwidth pulses, the center of the excitation profile curves downwards (Figure S5), and thus, an extra minor correction is performed with the normalized phase‐modulation $\phi_{\mathrm{norm}}\left(t\right)$ which peaks at the center of the frequency sweep. Due to the sequential tipping of spins in the frequency sweep, correcting the shape function of $B_{1x}^{\prime }\left(t\right)$ in this manner increases the RF amplitude experienced by spins distal to the coil.

## 3 Methods

### 3.1 Simulations

A Bloch simulator based on a 4th‐order Runge–Kutta solver was written in Python in the first rotating frame. Resilience to $B_{0}$ inhomogeneity was investigated by varying the Larmor frequency offset Ω. For all other simulations, unless noted otherwise, Ω = 0, *T*_1_ = *T*_2_ = 100 s (to effectively ignore relaxation), time step d*t* = 1e‐6 s, and $\omega_{1y}^{\prime }/2\pi$ varied from 0 to 10 000 Hz. Visualizations were written using custom Python and MATLAB scripts. Initial magnetization **M**_0_ is specified as a vector indicating the starting position of the magnetization along *x*′, *y*′, and *z*′ (formatted [*x*′ *y*′ *z*′ with origin [0 0 0]).

#### 3.1.1 Slice Selection With Two Coils

**FIGURE 3:**
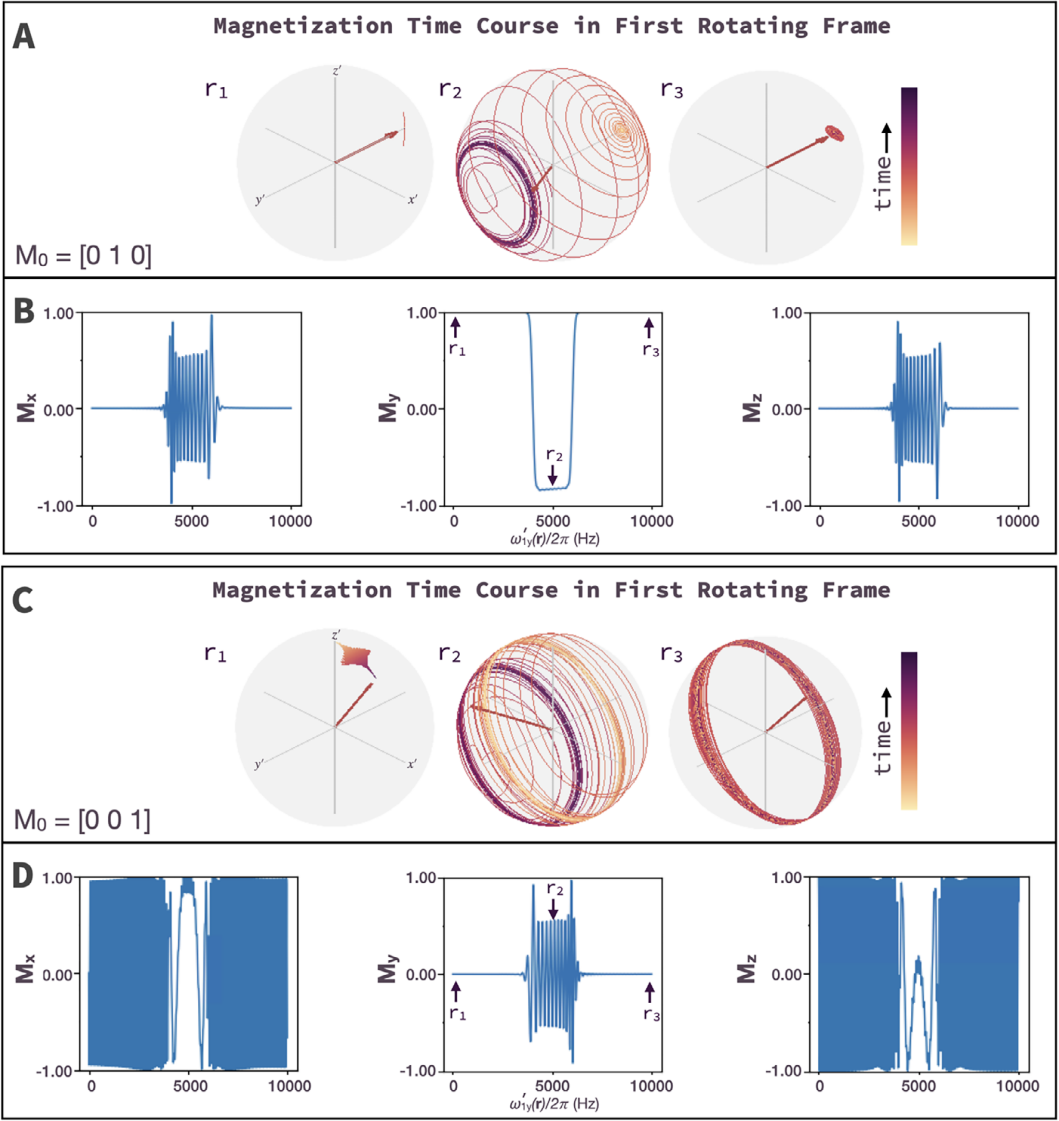
Magnetization trajectories and slice profiles produced by AM_HS1_. The magnetization (**M**(*t*)) in the first rotating frame is shown at three positions in a $B_{1y}^{\prime }$ gradient; r_1_ and r_3_ are far off resonance in the Rabi domain, and r_2_ is on resonance with the Rabi frequency at the center of the slice. (A) Trajectories for **M**_0_ = [0 1 0]. (B) Resulting **M**_x_, **M**_y_, and **M**_z_ sampled along the $B_{1y}^{\prime }$ gradient at the end of the pulse demonstrating nearly complete magnetization inversion (from +*y*′ to −*y*′). (C) Trajectories for the case of **M**_0_ = [0 0 1]. (D) Resulting **M**_x_, **M**_y_, and **M**_z_ sampled along the $B_{1y}^{\prime }$ gradient at the end of the pulse, demonstrating the presence of oscillation both inside and outside the slice.

A band of Rabi frequencies can be selectively inverted when $B_{1x}^{\prime }$ is an adiabatic HS1 pulse applied with a $B_{1y}^{\prime }$ gradient that generates a continuum of Rabi frequencies in the *x*′*z*′ plane. This simulation assumes two RF coils orthogonal to each other along the *x*‐ and *y*‐axes in the lab frame (Figure 3). Here, pulse parameters were chosen just below the threshold for full adiabaticity. Simulations of AM_HS1_ slice selection with two coils used the following settings: center Rabi frequency $\omega_{1y}^{\prime }\left(r_{\text{center}}\right)/2\pi =5000\;\mathrm{Hz}$, $\omega_{1x}^{\max}/2\pi =1000\;\mathrm{Hz}$, *T*_p_ = 10 ms, *R* = 20 (pulse bandwidth = 2000 Hz), and **M**_0_ = [0 1 0] or [0 0 1].

#### 3.1.2 Slice Selection With One Coil

Use of a single coil can facilitate the implementation of $B_{1}$‐selective methods and allow for their extension to parallel transmit arrays. Specifically, in the future parallel transmission should enable the synthesis of relatively linear RF gradients by allowing tailored weightings of the individual coil amplitudes. Regardless of whether the RF coil is a simple surface coil or a parallel transmit array, AM_HS1_ is synthesized from the $B_{1x}^{\prime }$ and $B_{1y}^{\prime }$ components of the two‐coil case (Equations (22), (23), (24)), but implemented as real and imaginary components of the (single) pulse. Simulations of slice inversion with a single coil used an AM_HS1_ pulse with $\omega_{1y}^{\prime }\left(r_{\text{center}}\right)/2\pi =5000\;\mathrm{Hz}$ (again $\omega_{1y}^{\prime }/2\pi$ is varied from 0 to 10 000 Hz), $\omega_{1x}^{\max}/2\pi =2000\;\mathrm{Hz}$, *T*_p_ = 10 ms, *R* = 20, and **M**_0_ = [0 1 0].

Simulations demonstrating sub‐adiabatic excitation ($\theta \leq 90^{o}$) using AM_HS1_ with one coil versus two coils were also performed (Figure 6) with: $\omega_{1y}^{\prime }\left(r_{\text{center}}\right)/2\pi =5000\;\mathrm{Hz}$, $\omega_{1x}^{\max}/2\pi =250\;\mathrm{Hz}$, *T*_p_ = 10 ms, *R* = 20, and **M**_0_ = [0 1 0].

Simulations were also performed to examine the slice profile for one coil when restricting the maximum RF amplitude, as in clinical MRI. These simulations assumed a $B_{1}$ gradient of $\omega_{1y}^{\prime }\left(r\right)/2\pi =500-3000\mathrm{Hz}$ (1000 steps), with $\omega_{1y}^{\prime }\left(r_{\text{center}}\right)/2\pi =1000\;\mathrm{Hz}$. The amplitude of the frequency‐swept component, $\omega_{1x}^{\max}/2\pi$, was varied from 0 to 3000 Hz. Increasing *R* values (2.5, 5, 7.5, 10, and 20) were evaluated. Other parameters were: *T*_p_ = 15 ms and **M**_0_ = [0 1 0].

To remove the linear and quadratic phase that results with a single coil when **M**_0_ = [0 0 1] and $\theta \leq 90^{o}$, a version of AM_HS1_ with $B_{1y}^{\prime }$ refocusing lobes was also investigated. The total refocusing lobe length was the same as the original $B_{1y}^{\prime }$ gradient pulse duration and was appended to both ends of the AM_HS1_ pulse. A two‐step phase cycle was performed by running the simulation again with negative phases and averaging the magnetization profiles of the two simulations. Subsequently the amplitude‐correction procedure described in Section 2.3 was implemented where the original shape function $F_{1}\left(t\right)$ was scaled by the normalized frequency sweep and phase modulation functions in Equation (30) for both phase cycle steps. Simulation parameters were: $\omega_{1y}^{\prime }\left(r_{\text{center}}\right)/2\pi =1000\;\mathrm{Hz}$, $\omega_{1x}^{\max}/2\pi =200\;\mathrm{Hz}$, *T*_p_ = 11.98 ms (including refocusing lobes), *R* = 10.4, **M**_0_ = [0 0 1], and an applied gradient $\omega_{G}^{\prime }/2\pi =0-5000\;\mathrm{Hz}$.

#### 3.1.3 Off‐Resonance and B_1_ Inhomogeneity Performance

Having established a method for slice selection, AM_HS1_ can be compared to other existing pulses such as a SLR‐optimized pulse. The latter is a derivative of Hoult's $B_{1}$‐selective imaging method and modulates a *z*′‐axis off‐resonance field in a frame rotating at $\omega_{0}+\Delta \omega_{\mathrm{RF}}\left(t\right)$ for slice selection [6]. The pulse shape of the z′‐axis off‐resonance field is optimized through the SLR algorithm and a $B_{1x}^{\prime }$ gradient is applied along the *x*′‐axis. Here, we have recreated their $B_{1}$‐selective SLR‐pulse to excite the same bandwidth as our AM_HS1_ pulse. Because AM_HS1_ is an FM pulse that is phase modulated in the *x*′*y*′ plane, the SLR pulse is instead formed similarly to ours with an *x*′‐and *y*′‐component rather than an *x*′‐and *z*′‐component. We then compare the behavior of both pulses when a constant resonance offset (Ω) is added along the *z*′‐axis to simulate $B_{0}$ inhomogeneity.

Adiabatic inversion with AM_HS1_ under increasing Ω was compared with a SLR‐optimized $B_{1}$‐selective pulse derived using the method of Grissom et al. [6]. For AM_HS1_, the following parameters were used: two coils, $\omega_{1y}^{\prime }\left(r_{\text{center}}\right)/2\pi =5000\;\mathrm{Hz}$, $\omega_{1x}^{\max}/2\pi =3000\;\mathrm{Hz}$, *T*_p_ = 10 ms, *R* = 30, and **M**_0_ = [0 1 0].

For the SLR pulse, a split and reflected pulse was used as indicated in Grissom et al., since θ > 90°. Simulation parameters were: two coils, $\omega_{1y}^{\prime }\left(r_{\text{center}}\right)/2\pi =5000\;\mathrm{Hz}$, *T*_p_ = 10 ms (two 5 ms segments), flip angle *θ* = 180°, *R* = 15, **M**_0_ = [0 0 1]. The *x*′‐axis RF component $\omega_{1x}^{\prime }/2\pi$ was stepped from 0 to 10 000 Hz.

These two pulses were compared under increasing $B_{1x}^{\prime }$ magnitude to demonstrate performance under inhomogeneous $B_{1x}$ and variable adiabaticity. The same settings were used for these simulations as above, however $\omega_{1x}^{\max}/2\pi$ was varied from 0 to 2926 Hz for AM_HS1_. Since the SLR algorithm can only use flip angle θ to change the applied $B_{1}$ amplitude, separate SLR pulses were generated for each of the 100 steps of the $B_{1}$ amplitude sweep from 0° to 360° corresponding to $\omega_{1x\_\mathrm{SLR}}^{\max}/2\pi$ of 0–2926 Hz.

### 3.2 Experimental Demonstration of AM_HS1_

Experimental data were acquired on a 9.4 T 31‐cm horizontal‐bore magnet with a Bruker ParaVision 3.5 console (Billerica, MA, USA) on phantoms and rats. A 2.8 cm diameter surface coil was used for imaging.

For slice selection in rat brain, 90° and 30° AM_HS1_ pulses were implemented with the addition of square $B_{1y}^{\prime }$ refocusing lobes and a two‐step phase cycle to reduce scan time. Settings used were: *R* = 1.21, *T*_p_ = 11.98 ms (including refocusing lobes), $\omega_{1x}^{\max}/2\pi =100\;\mathrm{Hz}$ (30°) or 400Hz (90°), $\omega_{1y}^{\prime }\left(r_{\text{center}}\right)/2\pi =2000\;\mathrm{Hz}$, *T*_R_ = 100 ms, *T*_E_ = 9.319 ms, FOV = 32 ×32 mm (256 × 256 pixels), scan time = 4 min 16 s, and RF power = 14 W. A coronal slice was obtained with 4 total averages across phase cycles, whereas axial slices were obtained with 10 total averages. This was compared to slice selection using $B_{0}$ gradients from a 2D gradient recalled echo (GRE) using a ∼90^o^ sinc pulse, and *T*_p_ = 5 ms, *T*_R_ = 100 ms, *T*_E_ = 13.329 ms, slice thickness = 1 mm, with the same FOV.

Slab selection was also demonstrated in vivo using a repurposed 3D GRE sequence. Specifically, the $B_{0}$ slab‐selective gradients were turned off and a 15° AM_HS1_ pulse with refocusing lobes and a two‐step phase cycle was used instead. Settings used were: 4 total averages, *R* = 10.4, *T*_p_ = 11.98 ms (including refocusing lobes), $\omega_{1x}^{\max}/2\pi$ = 200 Hz (15°), $\omega_{1y}^{\prime }\left(r_{\text{center}}\right)/2\pi =1000\;\mathrm{Hz}$, *T*_R_ = 50 ms, *T*_E_ = 7.857 ms, FOV = 32 × 32 × 20 mm (128 × 128 × 16, section thickness = 1.25 mm), scan time = 6 min 48 s, and RF power= 16.7 W. Due to the wide bandwidth and use of a single coil, the method for slice profile correction (Section 3.1.2) was also implemented which involved scaling the $B_{1x}^{\prime }\left(t\right)$ AM function $F_{1}\left(t\right)$ by the normalized frequency sweep and phase‐modulation functions.

This study was carried out in compliance with ARRIVE guidelines, and procedures were approved by the Institutional Animal Care and Use Committee of the University of Minnesota. A male Sprague–Dawley rat, 302 g and 68 days, and a female Wistar rat, 290 g and 94 days were anesthetized using isoflurane (5% for induction, then decreased to 2%) with O_2_/N_2_O (30%/70%) carrier gas. Respiration rate was monitored throughout the study. Temperature was monitored using a rectal thermometer and maintained at ∼37°C.

## 4 Results

### 4.1 Slice Selection With Two Coils

Magnetization trajectories were plotted over time for $\omega_{1y}^{\prime }/2\pi =10$, 5000, and 10 000 Hz, corresponding to low $B_{1y}^{\prime }$ (r_1_), the slice center (r_2_), and large $B_{1y}^{\prime }$ (r_3_), respectively (Figure 3). The results for spin inversion with two‐coils are straightforward for **M**_0_ = [0 1 0] because magnetization remains aligned with $\omega_{\mathrm{eff}}\left(t\right)$ as it is dragged from +*y*′ to −*y*′. In Figure 3A, the magnetization trajectories both inside and outside of the slice are shown. The slice is shown using the final **M**_y_ at the end of the AM_HS1_ pulse (Figure 3B). Magnetization vectors at r_1_ and r_3_ outside the slice move minimally from their initial position in time. For magnetization within the bandwidth of the pulse at r_2_, a slice is inverted ([0 1 0] ➔ [0 –1 0]). Conversely, due to non‐ideal adiabaticity, some magnetization components do not follow $\omega_{\mathrm{eff}}\left(t\right)$ and are left with a spread of phases in the *x*′*z*′ plane as indicated by the final **M**_x_ and **M**_z_ in Figure 3B.

When **M**_0_ = [0 0 1] (Figure 3C,D), the spins are not initially aligned with $\omega_{\mathrm{eff}}\left(t\right)$, and consequently, the resulting phase of the magnetization varies approximately quadratically in the slice and approximately linearly outside the slice as evidenced by the final **M**_x_ and **M**_z_ (Figure 3D).

### 4.2 Extending Slice Selection to One Coil

**FIGURE 4:**
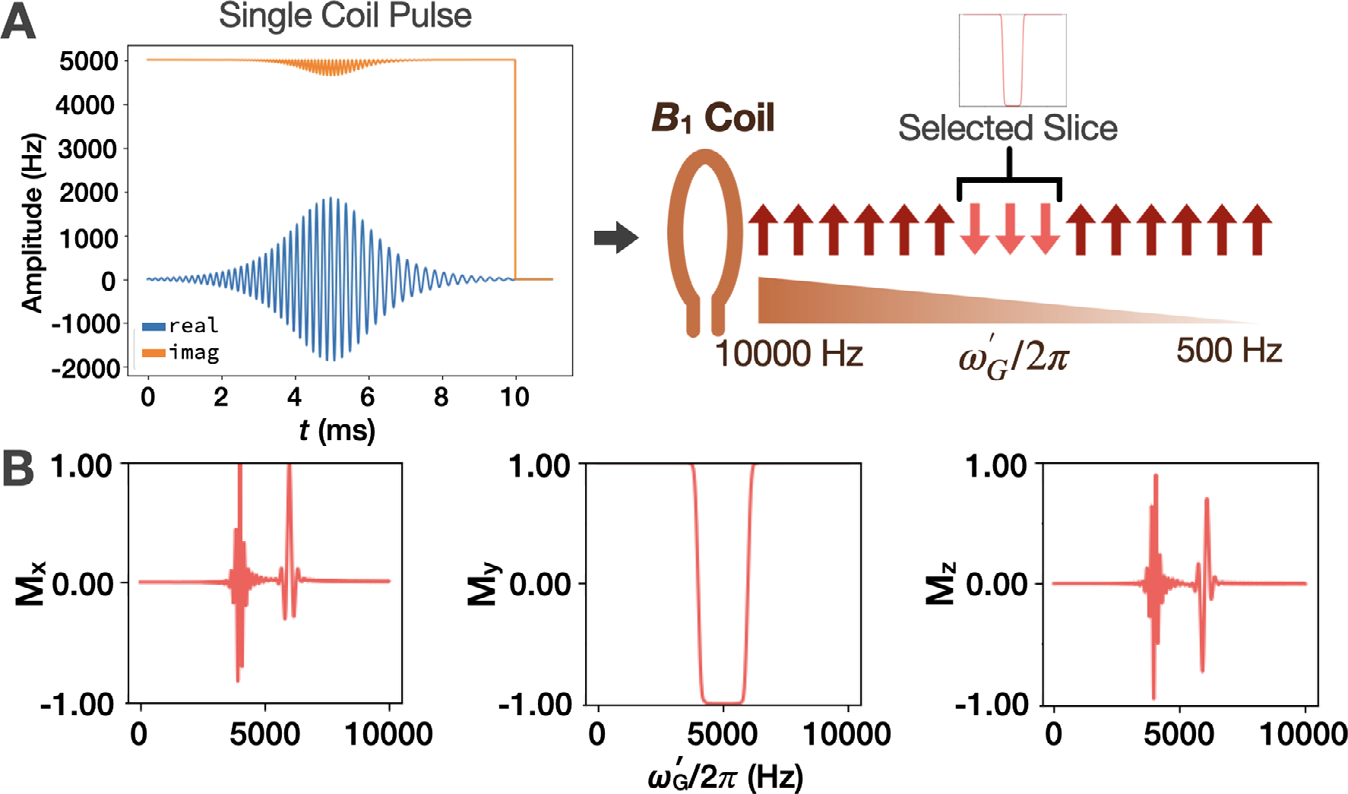
Slice selection with a single coil using AM_HS1_ pulse. (A) (Left) Real and imaginary components of AM_HS1_. (Right) The amplitude‐modulation of the real component produces a frequency sweep that inverts spins within its bandwidth. (B) **M**_*x*_, **M**_*y*_, and **M**_*z*_ sampled along the $B_{1}$ gradient at the end of the pulse demonstrating inversion from +*y*′ to −*y*′.

The process of making a constant amplitude AM_HS1_ by combining the two‐coil $B_{1x}^{\prime }\left(t\right)$ and $B_{1y}^{\prime }\left(t\right)$ results in a slight variation in the amplitude of $B_{\mathrm{Gy}}$, but the final pulse produces reasonable inversion for the selected slice when starting with **M**_0_ = [0 1 0] (Figure 4).

**FIGURE 5:**
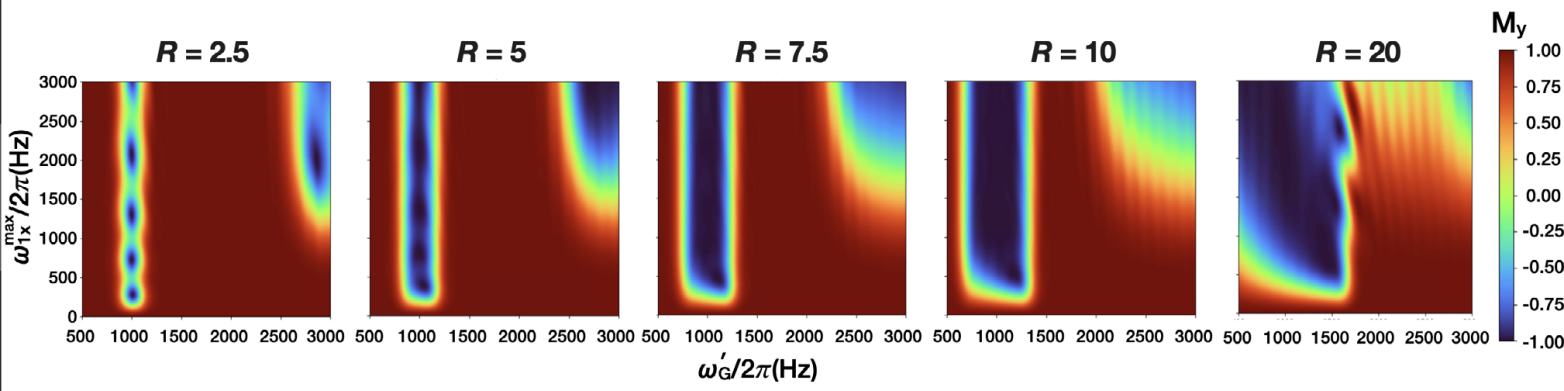
Slice inversion as a function of increasing $\omega_{1x}^{\max}/2\pi$ and increasing *R* value (time‐bandwidth product) for an AM_HS1_ pulse that is centered at $\omega_{1y}^{\prime }\left(r_{\text{center}}\right)/2\pi =1000\;\mathrm{Hz}$ and experiences a gradient of 500–3000 Hz, closer to the maximum RF amplitudes achievable with a head coil on a clinical MRI scanner.

For one coil, the ratio between $B_{1x}^{\prime }$ and $B_{1y}^{\prime }$ is important, especially for clinical scanners which may not have the range of RF powers simulated here. To qualitatively determine reasonable ranges for $B_{1x}^{\prime }$ and $B_{1y}^{\prime }$, the same simulations were repeated for increasing $B_{1x}^{\prime }$ using lower RF amplitudes closer to those achievable in clinical MRI (Figure 5). When used with $\omega_{1y}^{\prime }\left(r_{\text{center}}\right)/2\pi$ =1000 Hz, adiabaticity begins at *R* ≳ 7.5. At *R* = 20, the profile is degraded because the inversion bandwidth intersects $\omega_{G}^{\prime }/2\pi$ = 0 Hz. Lastly, for all *R* values, unwanted excitations at $\omega_{G}^{\prime }˜3\omega_{1y}\left(r_{\text{center}}\right)$ develop as $\omega_{1x}^{\max}$ increases, establishing an approximate minimum ratio for $B_{1y}^{\prime }$/$B_{1x}^{\prime }$ to be used when synthesizing AM_HS1_.

**FIGURE 6:**
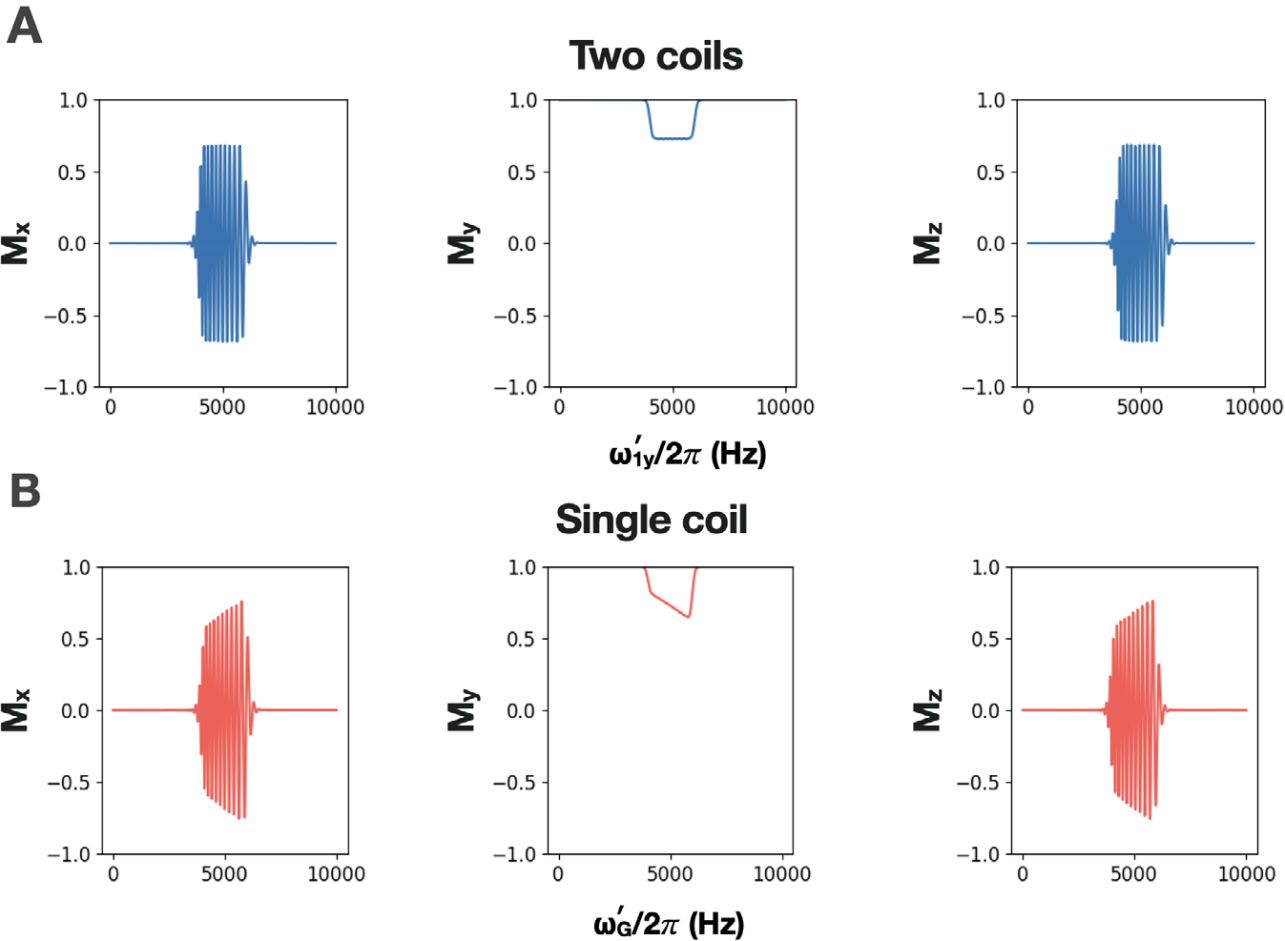
Comparison of excitation slice profiles generated by (A) two coils vs. (B) one coil. The simulation plots magnetization components **M**_*x*_, **M**_*y*_, and **M**_*z*_ at the end of a sub‐adiabatic 10 ms AM_HS1_ pulse with $\omega_{1x}^{\max}/2\pi =250\;\mathrm{Hz}$, *R* = 20, $\omega_{1y}^{\prime }\left(r_{\text{center}}\right)/2\pi =500\;\mathrm{Hz}$, and an applied $\omega_{1y}^{\prime }/2\pi$ or $\omega_{G}^{\prime }/2\pi$ gradient of 0–10 000 Hz.

As shown above, full adiabatic inversion can be achieved despite the presence of field inhomogeneity in the one‐coil case. However, when executing the pulse under sub‐adiabatic conditions, an uneven excitation profile is produced (Figure 6). To remedy this, the pulse must be scaled according to Equations ((28), (29), (30)).

**FIGURE 7:**
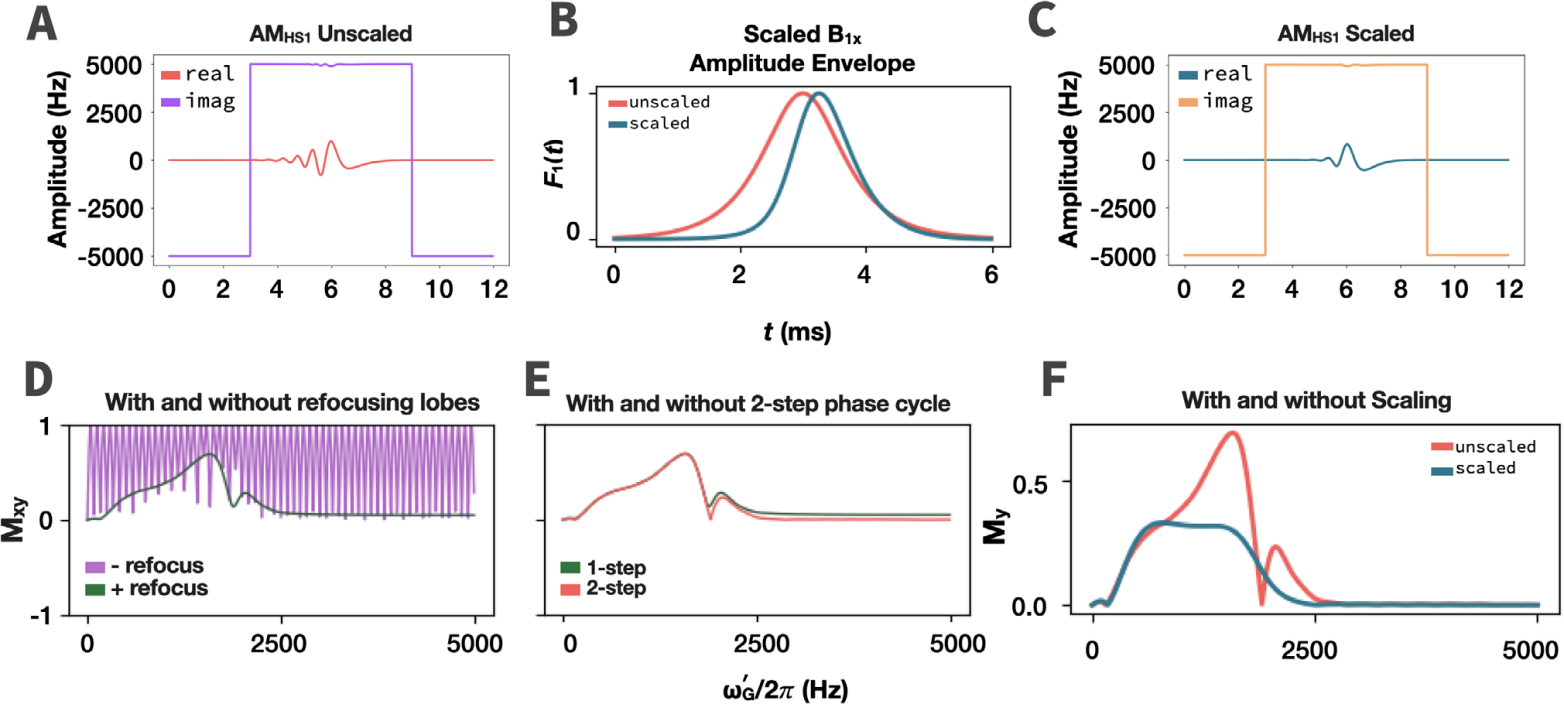
Excitation pulse shapes and magnetization profiles for low flip‐angle AM_HS1_ using a single coil, with and without refocusing lobes and RF scaling. (A) Original $B_{1x}^{\prime }$ and $B_{1y}^{\prime }$functions in time on a single coil. (B) Unscaled vs. scaled AM functions in time. The AM function, $F_{1}\left(t\right),$ is scaled by normalized frequency sweep and phase modulation functions such that spins at higher $B_{1}$ values receive less power and vice‐versa. (C) Scaled $B_{1x}^{\prime }$ and $B_{1y}^{\prime }$functions in time on a single coil. (D) Excitation profiles (**M**_xy_) with and without refocusing lobes. Refocusing lobes eliminate out‐of‐band oscillations for **M**_0_ = [0 0 1]; however, (E) a two‐step phase cycle is required to eliminate the dispersive component of the signal. (F) Further excitation profile flattening shown in **M**_y_ before and after scaling the AM function.

In addition, when **M**_0_ = [0 0 1] the phase of the final magnetization varies linearly and quadratically across space. To eliminate undesirable oscillations, one may apply refocusing lobes to both ends of the pulse with a two‐step phase cycle (Figure 7). The latter removes a dispersive component in the magnetization profile, but for large *R* values and a single coil, the slice profile still has some dependence on the $B_{1x}^{\prime }$ gradient. Therefore, a second correction is applied by scaling the shape function, $F_{1}\left(t\right)$, by a normalized version of the frequency sweep and phase‐modulation functions (${F_{1}}_{\mathrm{cor}}\left(t\right)=F_{1}\left(t\right)FM_{\mathrm{norm}}\left(t\right)\phi_{\mathrm{norm}}\left(t\right)$). This slice profile correction reduces the slice inhomogeneity from 33.5% to 4.1% over a 435 Hz bandwidth (at the height of the excitation profile).

### 4.3 Off‐Resonance and B_1_ Inhomogeneity Resilience

**FIGURE 8:**
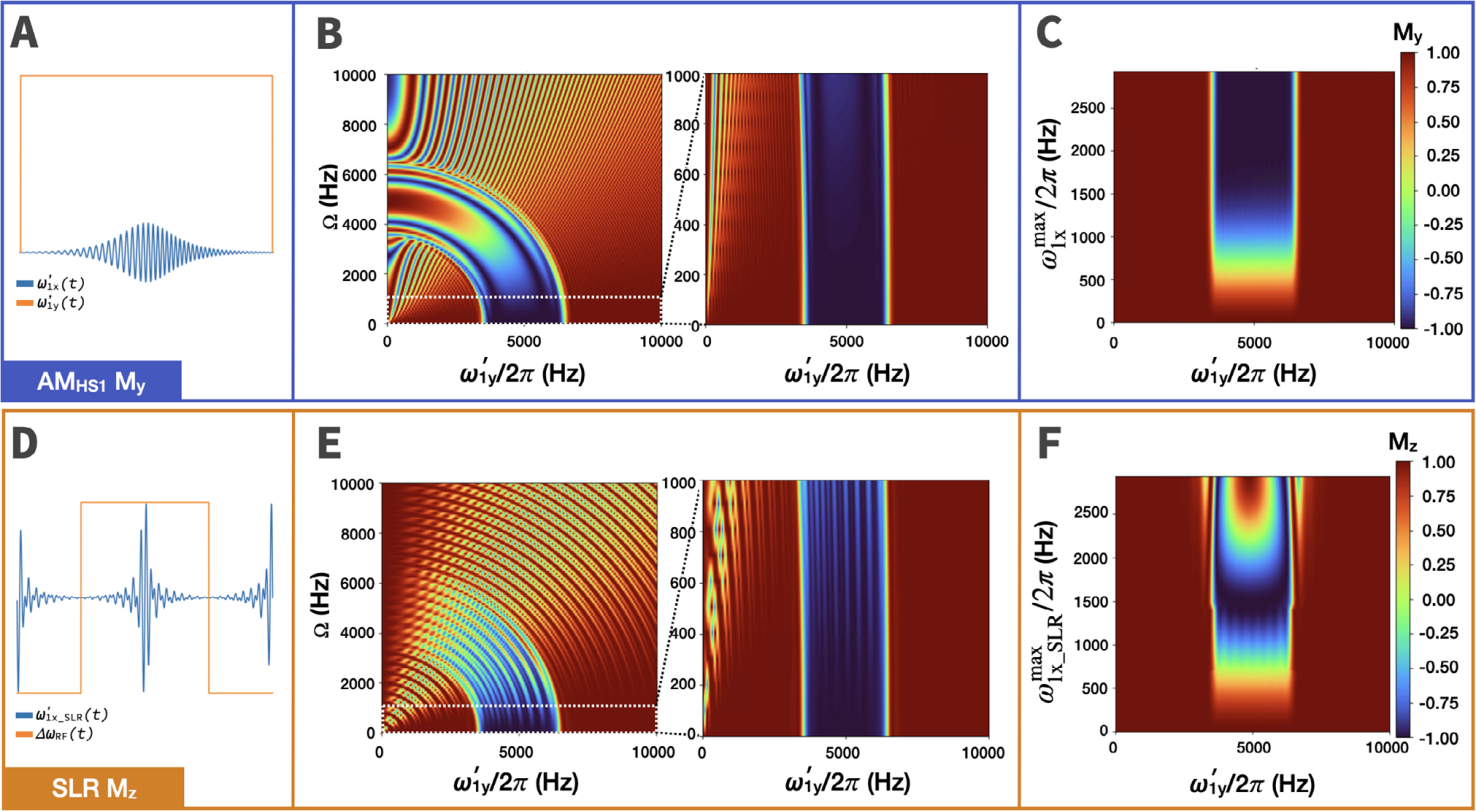
Comparison of inversion pulse profile for AM_HS1_ adiabatic $B_{1}$‐selective pulse vs. Shinnar‐Le‐Roux (SLR) $B_{1}$‐selective pulse for two RF coils. (A) AM_HS1_ pulse shape. (B) Off‐resonance performance for AM_HS1_ starting from **M**_0_ = [0 1 0]. (Left) Contour plot of **M**_y_ along a $B_{1y}^{\prime }$ gradient at the end of an AM_HS1_ pulse is shown as resonance offset increases. (Right) The same graph is shown but zoomed in (dotted outline) for offset range Ω = 0–1000 Hz. (C) Inversion pulse profile for AM_HS1_ as a function of increasing $\omega_{1x}^{\max}$ showing slice profile in the presence of $B_{1x}^{\prime }$ inhomogeneity. Similar graphs showing the (D) SLR pulse shape, (E) off‐resonance performance, and (F) inversion profile vs. $B_{1x}^{\prime }$ inhomogeneity for the SLR $B_{1}$‐selective pulse starting with **M**_0_ = [0 0 1].

With AM_HS1_ and SLR pulses the slice profile curves towards the off‐resonance direction with increasing Ω (Figure 8). For Ω = 0–1000 Hz, both AM_HS1_ and the SLR pulse achieve inversion, however the SLR pulse demonstrates more ripples in the inverted band. While only positive Ω values are shown, the inversion profile has reflection symmetry about Ω = 0.

Next, we compare the two pulses as the $\omega_{1x}^{\prime }$ amplitude increases, thus examining resilience to $B_{1x}^{\prime }$ inhomogeneity (Figure 8, right). AM_HS1_ is adiabatic and therefore maintains full inversion at Rabi frequencies above ∼1500 Hz, whereas the SLR pulse changes flip angle with increasing $\omega_{1x}^{\prime }$.

### 4.4 Experimental Demonstration of AM_HS1_

**FIGURE 9:**
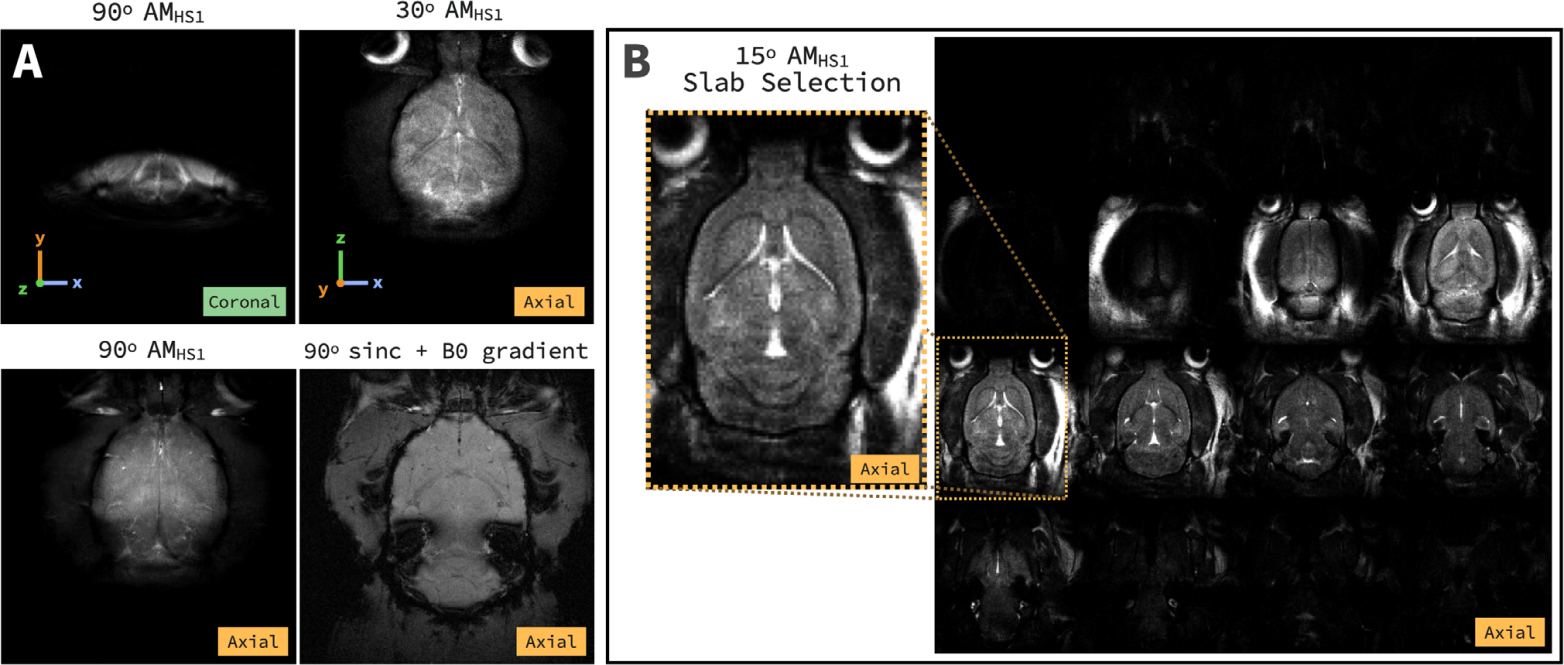
In vivo rat brain images using AM_HS1_ pulse for slice and slab selection. (A) Slice selection using 90° and 30° AM_HS1_ pulses with refocusing lobes, two‐step phase cycles and averaging showing coronal and axial views of rat brain with contrast highlighting cerebrospinal fluid (CSF) and vasculature. This is compared to slice selection using a 90° sinc pulse in a 2D GRE sequence. (B) Slab selection using 15° AM_HS1_ with slice profile correction instead of $B_{0}$ gradients and larger *R* value. (Left) Central slice shows stronger contrast of CSF and vasculature. (Right) Montage showing slab selectivity along the $B_{1}$ gradient in the FOV.

For in vivo rat brain imaging, slice selection using AM_HS1_ with refocusing lobes and a two‐step phase cycle (no AHP) achieved ∼1 mm slice thickness with *R* = 1.21 at $\omega_{1y}^{\prime }\left(r_{\text{center}}\right)/2\pi =2000\;\mathrm{Hz}$ (Figure 9A). Due to the small *R* needed for thin slice selection, slab selection was also demonstrated in vivo using *R* = 10.4 and $\omega_{1y}^{\prime }\left(r_{\text{center}}\right)/2\pi =1000\;\mathrm{Hz}$. This also used the method for slice profile correction outlined above.

## 5 Discussion

### 5.1 Slice Selection With Two Coils

Simulations for $B_{1}$‐based slice selection using AM_HS1_ with two RF coils show distinct slices at the expected locations starting with **M**_0_ along the *y*′‐and *z*′‐axes (Figure 3). For **M**_0_ along the *z*′‐axis, a series of oscillations are superimposed over the slice. While undesirable, these oscillations may be canceled by refocusing lobes (Figure 7). Without refocusing lobes, slice selection starting with **M**_0_ = [0 0 1] requires a preparatory non‐selective 90° pulse prior to AM_HS1_, as done in phantom imaging (Figure S3). Overall, these results demonstrate the utility of adiabatic pulses for $B_{1}$‐selective slice inversion and refocusing.

### 5.2 Extending Slice Selection to One Coil

Slice inversion is still accomplished well when moving to one coil, however now the pulse profile varies with position if the pulse is not applied with sufficient power to produce full adiabatic inversion (Figures 6 and 7) because the amplitude of $B_{1x}^{\prime }$ depends on position. Although the magnetization phases in the *x*′*z*′ plane are notably different from the two‐coil case, they remain quadratic as evidenced by the envelope of **M**_x_ and **M**_z_. The maximum pulse amplitude, $\omega_{1x}^{\max}/2\pi$, must be increased from 1000 Hz (two‐coil) to ∼1414 Hz (one coil) to maintain inversion. This is likely a consequence of how the pulse was designed to maintain a constant total amplitude, $B_{G}$. That is, $B_{1x}^{\prime }\left(t\right)$ increases at the center of the pulse at the expense of $B_{1y}^{\prime }\left(t\right)$. In the future, improvements in performance can be expected with alternative pulse design strategies, including allowing $B_{G}$ to be time dependent.

Simulations show that a large $B_{1y}^{\prime }:B_{1x}^{\prime }$ ratio is key to avoiding unwanted excitations. In Figure 5, slice position, $\omega_{1y}^{\prime }\left(r_{\text{center}}\right)/2\pi$, is set to a low value (1000 Hz), while $\omega_{1x}^{\max}/2\pi$ is varied between 500 and 3000 Hz to emulate $B_{1}$ amplitudes expected to be practically achievable with a low‐ or mid‐field scanner. Pulses with low *R* < 5 do not produce an even slice profile or behave adiabatically. *R* ≳ 7.5 is needed for adiabaticity; however, at *R* > 10, the pulse profile starts degrading. This is likely from the interference of a separate wider slice at approximately three times the center frequency when large $\omega_{1x}^{\max}$ values are used. This interference is most apparent at *R* = 20 and $\omega_{G}^{\prime }/2\pi$≈1500 Hz where the slice profile degrades as the two excitation bands overlap. As the *R* value increases at fixed pulse duration, the Rabi frequencies in the sweep extend to both lower to higher values. As the sweep includes more low frequencies, the rotating wave approximation becomes more difficult to satisfy, and as a result, the slab profile deteriorates. This was also seen in an SLR‐optimized version of Bloch‐Siegert imaging [7], and was explained as multiphoton resonances. Hoult noted that $B_{1}$‐selective imaging only maintains selectivity when $B_{2}\ll B_{1}$, aka $B_{1x}^{\prime }\ll B_{1y}^{\prime }$. The extent of tolerable out‐of‐band excitations may vary, but our rule‐of‐thumb would be to use $B_{1y}^{\prime }{>2B}_{1x}^{\prime }$_._ While the precise mechanism for these excitations is unclear, we believe this represents a violation of the rotating wave approximation [16]. Yet, these simulations show that AM_HS1_ is feasible when using $B_{1}$ amplitudes accessible to clinical MRI given $B_{1y}^{\prime }$:$B_{1x}^{\prime }$ is set properly.

A side effect that arises when moving to a single coil is that, for sub‐adiabatic pulses below the $\omega_{1x}^{\max}/2\pi =1414\;\mathrm{Hz}$ threshold, the excitation profile is quadratic rather than flat (Figure 6, one coil). In the two‐coil case, $B_{1x}^{\prime }$ is homogenous; however in the one‐coil case $B_{1x}^{\prime }$ forms a linear gradient. While this does not affect inversion, pulses with flip angle <180° produce an uneven profile. To correct for this, AM_HS1_ can be applied with $B_{1y}^{\prime }$ refocusing lobes and a two‐step phase cycle (Figure 7). The phases for this refocused pulse must be carefully set such that the phase at the center of the pulse = ±90°. Despite phase‐cycling, some dependence on $B_{1x}^{\prime }$ remains, but this is compensated for by scaling by $FM_{\mathrm{norm}}\left(t\right)$ and $\phi_{\mathrm{norm}}\left(t\right)$. Another method for correcting the slice profile entails using AM_HS1_ in a BIR‐4 format [19], however it produces an unexpected out‐of‐band excitation at ${3\omega }_{1y}^{\prime }\left(r_{\text{center}}\right)$ (Figure S2). Notably, both methods generate a flat slice profile despite the $B_{1}$ inhomogeneity of the coil. The ability to acquire a slab or slice with an even profile at low flip angle is advantageous. A particularly interesting possibility for the future is excitation with uniform flip angle irrespective of coil geometry (so long as the approximate range of $B_{1}$ inhomogeneities is known). In particular, this could be a method for uniform low flip angle excitation with inhomogeneous RF coils, without needing to map $B_{1}^{+}$.

### 5.3 Off‐Resonance and B_1_ Inhomogeneity Performance

The off‐resonance performance of AM_HS1_ and slice uniformity with increasing $\omega_{1x}^{\max}$ were examined in comparison to an established method of $B_{1}$‐selective slice selection using the SLR algorithm (Figure 8). Both pulses produce profiles that curve towards the off‐resonance axis with increasing Ω, but for ∣Ω| < 1000 Hz the curvature is minimal. The SLR pulse demonstrates more ripples in the slice profile with larger ∣Ω| (Figure 8C). Grissom et al. examined their SLR pulse over an offset range of 0–1000 Hz, and this range is also shown here for both pulses.

As expected, AM_HS1_ behaves adiabatically, in that above a threshold $\omega_{1x}^{\prime }$, the slice profile remains nearly unchanged (Figure 8B). The SLR pulse, which closely resembles a sinc pulse formatted similarly to BIR‐4, is not adiabatic and therefore does not maintain inversion with increasing $B_{1x}^{\prime }$ amplitude (Figure 8D). Similarly, the SLR pulse is not adiabatic and exhibits many unwanted excitations as flip angle increases [6]. Overall, AM_HS1_ seems to provide the best resilience to resonance offset and $B_{1x}^{\prime }$ inhomogeneity with two coils.

### 5.4 Experimental Demonstration of AM_HS1_

Slice selection using only $B_{1}$‐gradients was demonstrated experimentally on both test tube and spherical water phantoms (Figure S3). Some out‐of‐band coherences occur for both; however, a four‐segment EXORCYCLE [17] removes these and produces a relatively flat slice profile.

AM_HS1_ using refocusing lobes and a two‐step phase cycle was also demonstrated in vivo to perform slice and slab selection in rat brain. Our initial experiment focusing on slice selection required a very small *R* = 1.21 to select a slice ∼1 mm in thickness (Figure 9A). While this produced interesting contrast, the pulse was no longer adiabatic due to the small *R*. Slab selection with AM_HS1_ with a higher *R* = 10.4 was also tested. Turning off the slab‐select $B_{0}$ gradients allowed for good slab selection using only AM_HS1_ (Figure 9B). The differences between the slice‐ and slab‐selected AM_HS1_ images and a typical B_0_‐selected GRE image are notable. AM_HS1_ produces stronger contrast of CSF and vasculature, likely related to a combination of *T*_1_ and *T*_2*ρ*_ relaxation. Muscle signal is significantly lowered in AM_HS1_ perhaps indicating sensitivity to magnetization transfer. The signal in the vitreous humor is increased at 30° compared to 90° which indicates *T*_1_ weighting as well [18].

Due to their high average RF power, $B_{1}$‐selective pulses, including AM_HS1_, are likely to be limited to human scanning at low‐ to mid‐field strengths. 9.4 T imaging was performed here just to demonstrate feasibility; however, the eventual goal is implementation on a mid‐field scanner such as our 0.7 T head‐only system [20]. Full‐wave 3D EM simulations [21] $B_{1}^{+}$ and SAR (Figure S4) demonstrate that a single curved double‐loop coil can produce the same ${\gamma B}_{1}^{+}/2\pi$ attained at 9.4 T of 1000 Hz approximately halfway into the head at 0.7 T. This will allow for slice selection of deep brain using AM_HS1_. Furthermore, the average and peak 10 g SAR meet FDA and IEC 60601‐2‐33:2022 limitations [22, 23] at 0.7 T, and thus, indicate the potential of performing human scans safely with the pulse in the future. Further improvements in SAR would involve the addition of a z‐coil tuned to the Rabi frequency of interest. This would create a circularly‐polarized field in the *x*′z′‐plane and would reduce unwanted excitations outside of the band of interest, allowing for lower $B_{1}^{+}$ to be used for slice selection. Furthermore, accounting for inter‐subject variability through a diverse human models' population can enhance the accuracy of SAR estimations and RF safety limitations.

## 6 Conclusion

In summary, we have introduced a $B_{1}$‐selective adiabatic pulse, AM_HS1_, that can perform slice and slab selection using only the $B_{1}$ gradient of a single RF coil having arbitrary geometry. The pulse produces a sharply demarcated inversion profile and is relatively insensitive to $B_{0}$ and $B_{1}$ inhomogeneities, although some sensitivity to $B_{0}$ still occurs at low $B_{1}$ values. To the best of our knowledge this is first time a shaped pulse has successfully been used for $B_{1}$‐selective excitation of a slice and slab in vivo. We hope these pulses can contribute to the repertoire of sequences being worked on for low‐cost/compact MRI scanners with $B_{1}$ instead of $B_{0}$ gradients. Ideally these spatially‐selective pulses will provide a foundation for a rich palette of imaging methods and contrasts on lower cost, compact MRI systems, without sacrificing image quality. Finally, the proposed pulse design may open new possibilities for creating novel tissue contrasts based on Rabi frequencies in high‐rank rotating frames.

## Funding

This work was supported by the National Institutes of Health, F30 DK137445, P41 EB027061, R56 EB033347, S10 OD032192.

## Conflicts of Interest

Efraín Torres is CEO of Adialante. The remaining authors have no conflicts of interest to disclose.

## Supporting information


**Figure S1.** A time‐varying amplitude‐modulation is equivalent to a frequency sweep. (A) For illustration, the effect of amplitude‐modulating a chirp pulse in the xy‐plane is shown. (B) Amplitude‐modulations occurring early and later in the chirp, within a constant time interval Δt. (C) Chirp pulse segments can be decomposed into two counterrotating fields rotating at a variable amplitude‐modulation frequency. The instantaneous vector amplitudes at time Δt are ±ω with blue vectors indicating the initial position and red vectors indicating the final position within the sampled Δt. Note, the higher amplitude‐modulation frequency (left) produces the higher absolute modulation frequency in the transverse plane
**Figure S2.** Demonstration of AM_HS1_ as a BIR‐4 pulse at varying flip angles using a single coil, two coils, and two coils plus a z‐coil. (A) Amplitude function, $F_{1}\left(t\right)$, alongside BIR‐4 phase functions, $\phi_{\mathrm{BIR}-4}\left(t\right)$ and $\phi_{\text{offset}}\left(t\right)$. The phase jumps occurring at *T*_p_/2 and 3T_p_/2 in $\phi_{\mathrm{BIR}-4}\left(t\right)$ determine the flip angle. The phase ramps in $\phi_{\text{offset}}\left(t\right)$ are flipped for the reflected AM_HS1_ segments. (B) Magnetization components *M*_*xy*_ sampled along the $B_{1}$ gradient at the end of the BIR‐4 AM_HS1_ pulse. Excitation with a single coil (red), two coils (blue), and two coils plus a *z*‐coil (green) is shown for flip angles 90° and 10°. An off‐resonance excitation is seen at 3 times the center frequency of the pulse that significantly reduces with two coils and is eliminated with the addition of a z‐coil that produces a field component that is 90° out of phase with $B_{1x}$
**Figure S3.** Experimental results of slice selection using an AM_HS1_ pulse and a surface coil on phantoms. (A) Pulse sequence for AM_HS1_ applied as a double spin echo. Phase encode (G_PH_) and readout (G_RO_) gradients are used purely for slice visualization. (B) Slice selection with a surface coil (arrow, cross‐section) placed at the center of a tube with slices on either end. (C) Slice selection with a surface coil placed on the left side of the sphere shown. (Left) Image after 4‐ms AHP. (Middle) Image after 4‐ms AHP + two consecutive AM_HS1_. (Right) Image after 4‐ms AHP + two consecutive AM_HS1_ using four‐step EXORCYCLE. Relative image intensity along dotted red line is shown below each image
**Figure S4.** CST Studio 3D full‐wave EM simulations of SAR and $B_{1}^{+}$ at 0.7 T. (A) Positioning of double‐loop coil in the simulation setup with dimensions and RF input port labeled. The coil was driven with 150 W incident RF power and a 20% duty cycle. (B) Simulation results of (Left) 10 g SAR and (Right) |$B_{1}^{+}$| through slices of the head
**Figure S5.** Comparison of low flip angle slice profile for AM_HS1_ using a single coil with (+phs) and without (−phs) phase modulation shape function scaling for (A) $\omega_{1y}^{\prime }\left(r_{\text{center}}\right)/2\pi =1000\mathrm{Hz}$ and (B) $\omega_{1y}^{\prime }\left(r_{\text{center}}\right)/2\pi =5000\;\mathrm{Hz}$. Both of these excitations use the FM shape function scaling.

## Data Availability

The data that support the findings of this study are available from the corresponding author upon reasonable request.
