# Supplementary material for: Designing B1 ‐Selective Pulses by Frequency Modulating in a Second Rotating Frame
Source: Magn Reson Med. 2026 Jan 18;95(5):2689–701. doi: 10.1002/mrm.70259 (PMC12962226; doi:10.1002/mrm.70259)
Supplement: Supplementary file 1 — Figure S1. A time‐varying amplitude‐modulation is equivalent to a frequency sweep. (A) For illustration, the effect of amplitude‐modulating a chirp pulse in the xy‐plane is shown. (B) Amplitude‐modulations occurring early and later in the chirp, within a constant time interval Δt. (C) Chirp pulse segments can be decomposed into two counterrotating fields rotating at a variable amplitude‐modulation frequency. The instantaneous vector amplitudes at time Δt are ±ω with blue vectors indicating the initial position and red vectors indicating the final position within the sampled Δt. Note, the higher amplitude‐modulation frequency (left) produces the higher absolute modulation frequency in the transverse plane Figure S2. Demonstration of AMHS1 as a BIR‐4 pulse at varying flip angles using a single coil, two coils, and two coils plus a z‐coil. (A) Amplitude function, F1(t), alongside BIR‐4 phase functions, ϕBIR−4(t) and ϕoffset(t). The phase jumps occurring at T p/2 and 3Tp/2 in ϕBIR−4(t) determine the flip angle. The phase ramps in ϕoffset(t) are flipped for the reflected AMHS1 segments. (B) Magnetization components M xy sampled along the B1 gradient at the end of the BIR‐4 AMHS1 pulse. Excitation with a single coil (red), two coils (blue), and two coils plus a z‐coil (green) is shown for flip angles 90° and 10°. An off‐resonance excitation is seen at 3 times the center frequency of the pulse that significantly reduces with two coils and is eliminated with the addition of a z‐coil that produces a field component that is 90° out of phase with B1x Figure S3. Experimental results of slice selection using an AMHS1 pulse and a surface coil on phantoms. (A) Pulse sequence for AMHS1 applied as a double spin echo. Phase encode (GPH) and readout (GRO) gradients are used purely for slice visualization. (B) Slice selection with a surface coil (arrow, cross‐section) placed at the center of a tube with slices on either end. (C) Slice selection with a surface coil placed on the left si [file MRM-95-2689-s001.docx]

**Supporting Information**


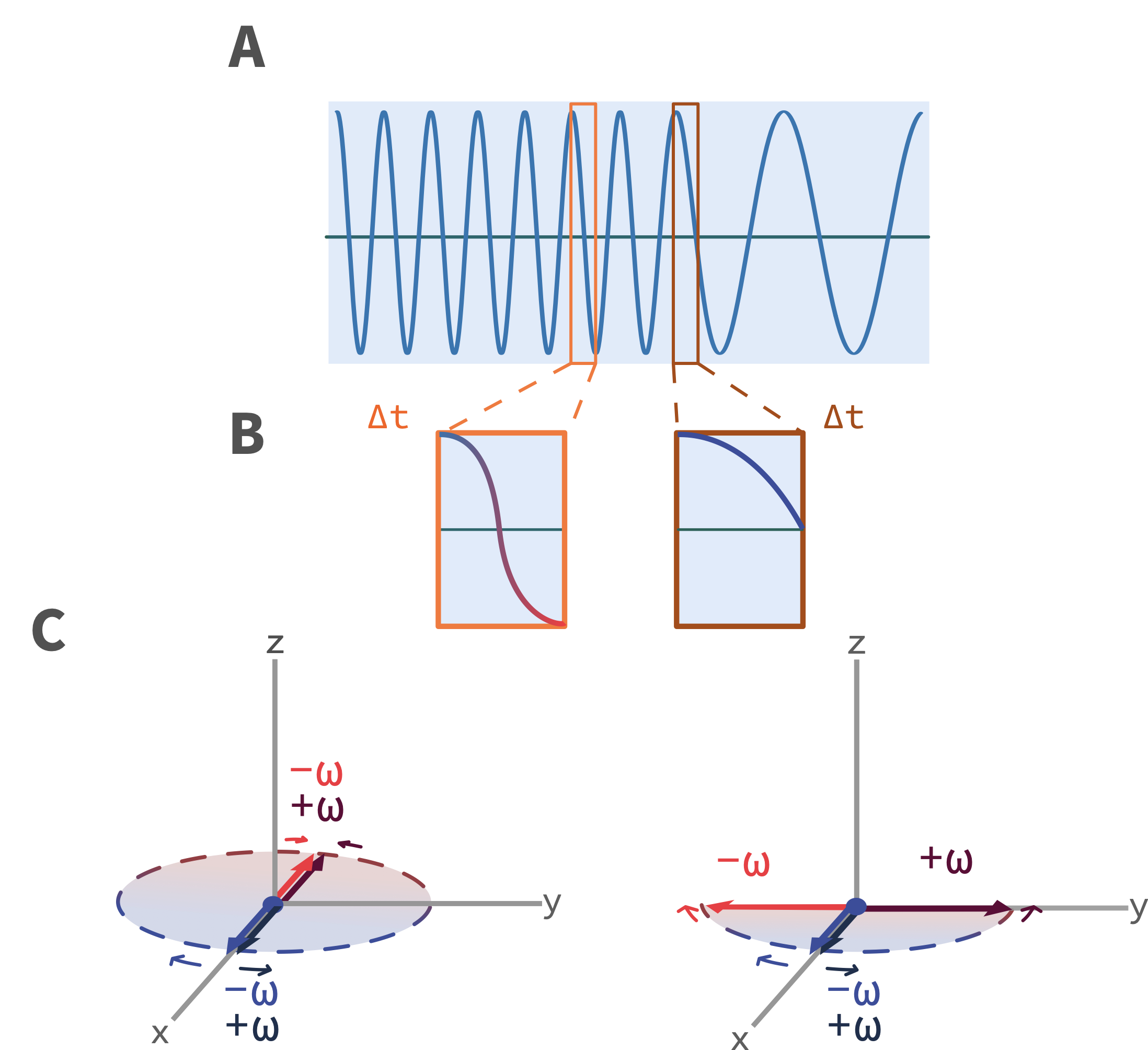


***Figure S1****. A time-varying amplitude-modulation is equivalent to a frequency sweep.* ***A)*** *For illustration, the effect of amplitude-modulating a chirp pulse in the xy-plane is shown.* ***B)*** *Amplitude-modulations occurring early and later in the chirp, within a constant time interval* $\Delta t$*.* ***C)*** *Chirp pulse segments can be decomposed into two counterrotating fields rotating at a variable amplitude-modulation frequency. The instantaneous vector amplitudes at time* $\Delta t$*are* $\pm\omega$ *with blue vectors indicating the initial position and red vectors indicating the final position within the sampled* $\Delta t$*. Note, the higher amplitude-modulation frequency (left) produces the higher absolute modulation frequency in the transverse plane.*

***Correction of Single Coil Slice Profile with BIR-4***

By operating below the threshold for adiabaticity, it is possible to produce flip angles < 180^o^, although the sensitivity to $B_{1}$ inhomogeneity returns, as with conventional sub-adiabatic HS1 pulses in the first rotating frame. When transmitting AM_HS1_ with a single coil under sub-adiabatic conditions, the flip angle in slice will vary in proportion to $B_{1x}^{'}\left( \mathbf{r} \right)$. Furthermore, when starting from M_0_ = [0 0 1], oscillations with frequency proportional to $\omega_{1y}^{'}\left( \mathbf{r} \right)$appear outside of the selected slice. Here, a method is represented to flatten this profile using BIR-4^[19]^. Simulations were performed to analyze its effectiveness **(Figure. S2)**.

For this conversion to BIR-4, the amplitude and phase functions for AM_HS1_ must be split and reflected on either end of an AM_HS1_ pulse^[19]^. Phase shifts are then applied before and after the middle pulse to determine the flip angle:

$$\begin{aligned} {\Delta\phi}_{BIR-4}=\pi+\frac{\theta}{2}\#\left( seq equation 18 \right) \end{aligned}$$

Phases at the points of highest $B_{1x}^{'}$ (t = 0, *T*_p_, and 2*T*_p_) must be carefully set such that they are close to either $\pm\frac{\pi}{2}$ (without phase shifts) to ensure a flat magnetization profile. This ensures maximum $B_{1x}^{'}$ at the center of the slice to produce a frequency sweep that is symmetric. The phase ramps used to center the slice can then be split and reflected in a similar manner and flipped in sign.

Simulation parameters for BIR-4 were: $\omega_{1y}^{'}\left( r_{\mathrm{center}} \right)/2\pi$ = 2500 Hz, $\omega_{1x}^{\max}/2\pi$ = 1500 Hz, *T*_p_ = 11.98 ms (including the refocusing lobes), *R* = 5.75, $\theta$ = 90^o^ and 10^o^, M_0_ = [0 0 1] and d*t* = 1e-7 s. These simulations were performed for a single coil, two coils, and two coils plus a z-coil that assumes the same field as $B_{1x}^{'}$ although 90^o^ out of phase to produce a circularly polarized x’z’ field.

This pulse can produce flat slice profiles at low flip angles when starting with M_0_ aligned with the z-axis as is typical. While the phases must be carefully set to ensure a symmetric pulse, flat excitation profiles are clearly shown for flip angles of 90^o^ and 10^o^ **(Figure. S2)**. An unexpected side effect of this was that a separate, wider bandwidth, lower flip angle excitation at 3 times the center frequency appears, as mentioned earlier. This is possibly related to the ratio of $B_{1x}^{'}$:$B_{1y}^{'}$ being too high, as this issue dramatically reduces with the presence of two coils where the $B_{1x}^{'}$ component no longer is a gradient. This issue can also be reduced by utilizing longer pulses, however this is generally undesirable due to SAR concerns. The addition of field along the z’-axis, 90º out of phase with $B_{1x}^{'}$ to produce a circularly polarized x’z’-field further reduces the out-of-band excitation. This could be included as a coil resonating in the kHz range along z.


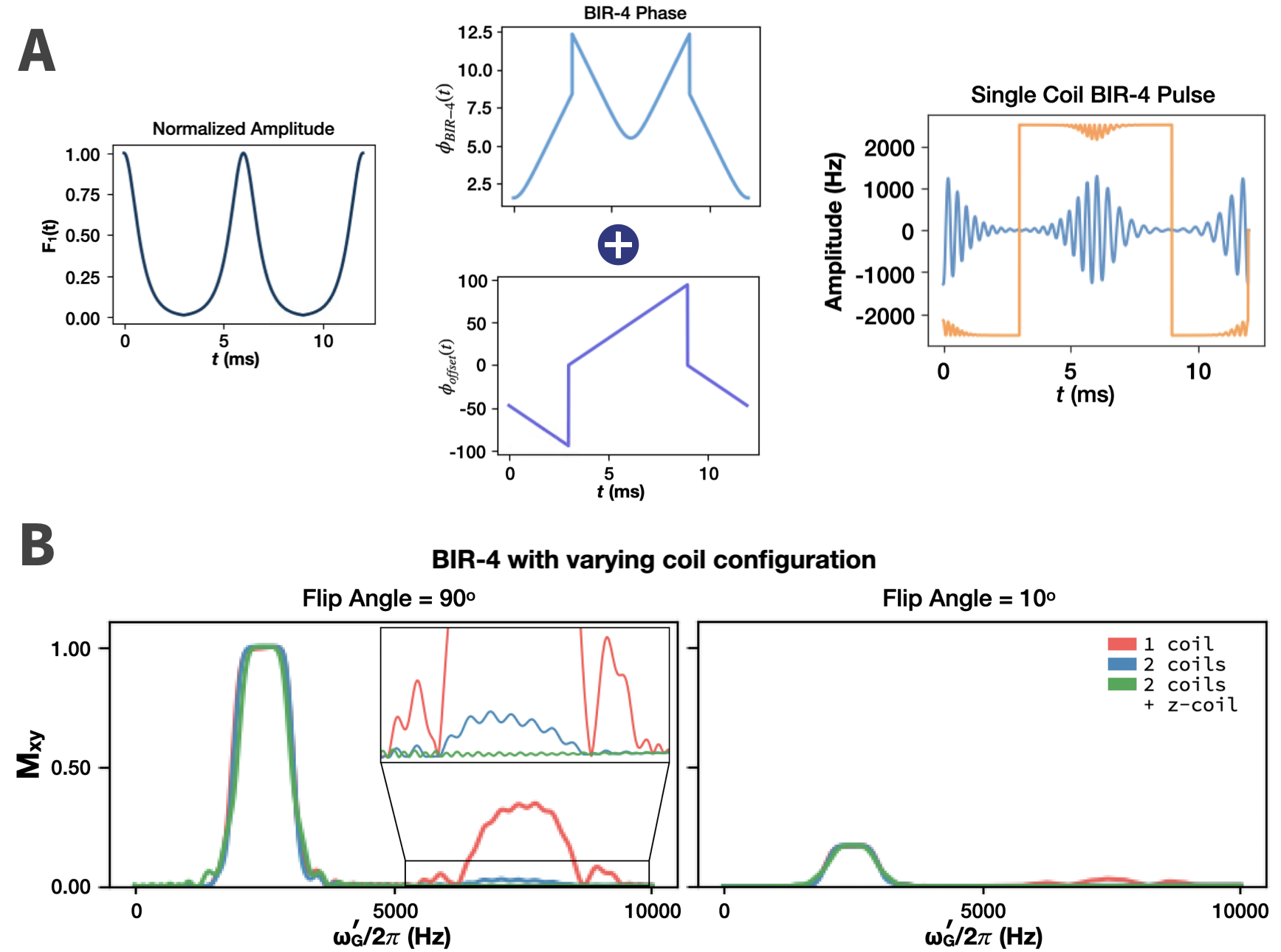


***Figure S2.*** *Demonstration of AM_HS1_ as a BIR-4 pulse at varying flip angles using a single coil, two coils, and two coils plus a z-coil.* ***A)*** *Amplitude function,* $F_{1}\left( t \right)$, *alongside BIR-4 phase functions,* $\phi_{BIR-4}\left( t \right)$ *and* $\phi_{\mathrm{offset}}\left( t \right)$*. The phase jumps occurring at T*_p_*/2 and 3T*_p_*/2 in* $\phi_{BIR-4}\left( t \right)$ *determine the flip angle. The phase ramps in* $\phi_{\mathrm{offset}}\left( t \right)$ *are flipped for the reflected AM_HS1_ segments.* ***B)*** *Magnetization components M_xy_ sampled along the* $B_{1}$ *gradient at the end of the BIR-4 AM_HS1_ pulse. Excitation with a single coil (red), two coils (blue), and two coils plus a z-coil (green) is shown for flip angles 90^o^ and 10^o^. An off-resonance excitation is seen at 3 times the center frequency of the pulse that significantly reduces with two coils and is eliminated with the addition of a z-coil that is 90^o^ out of phase with* $B_{1x}$*.*

**Experimental Demonstration of AM_HS1_ in Phantoms**

Slice selection using AM_HS1_ in a double spin-echo (DSE) sequence was demonstrated experimentally on a tube and a spherical water phantom at 9.4T using only surface coils **(Figure S3)**. Phantoms contained deionized (DI) water with 50 mM NaCl and 1% CuSO_4_. Surface coils having 1.5-cm and 3-cm diameters were used to image a 0.6-cm diameter tube and a 3.8-cm diameter sphere, respectively. The tube was placed symmetrically through the plane of the coil.

The pulse sequence uses a 4-ms adiabatic half passage (AHP) pulse to initialize all spins along the y’-axis and subsequently executes an 8-ms AM_HS1_ DSE sequence (two 4-ms AM_HS1_ pulses back-to-back) (**Figure S3A**). $B_{0}$-gradient encoding in the orthogonal directions was used purely to visualize the $B_{1}$-selected slice. Slice position was adjusted by changing RF power or $\phi_{\mathrm{off}}(t)$. Sequence settings were: $\omega_{1x}^{\max}/2\pi$ = 2000 Hz (sphere), 1000 Hz (tube); $\omega_{1y}^{'}(r_{\mathrm{center}})/2\pi$= 5000 Hz (sphere), 2000 Hz (tube), the largest value of $\omega_{G}^{'}/2\pi$ = 10000 Hz, *T*_R_ = 100 ms, *T*_E_ = 6.828 ms (from DSE center), RF power = 9.9 W (tube), 54.8 W (sphere). To remove out-of-band coherences, a four-segment Exorcycle^[17]^ was performed by changing the initial phase of the 2^nd^ AM_HS1_ pulse to 0^o^, 90^o^, 180^o^, and 270^o^ and combining the resulting slices accordingly (0^o^ slice + 180^o^ slice) - (90^o^ slice + 270^o^ slice).

In the tube image, two slices are visible **(Figure S3B)** because the $B_{1}$ profile is symmetric about the plane of the coil. A curved 3D slice is excited in the sphere which is further localized after using a four-segment EXORCYCLE **(Figure S3C)**.

***
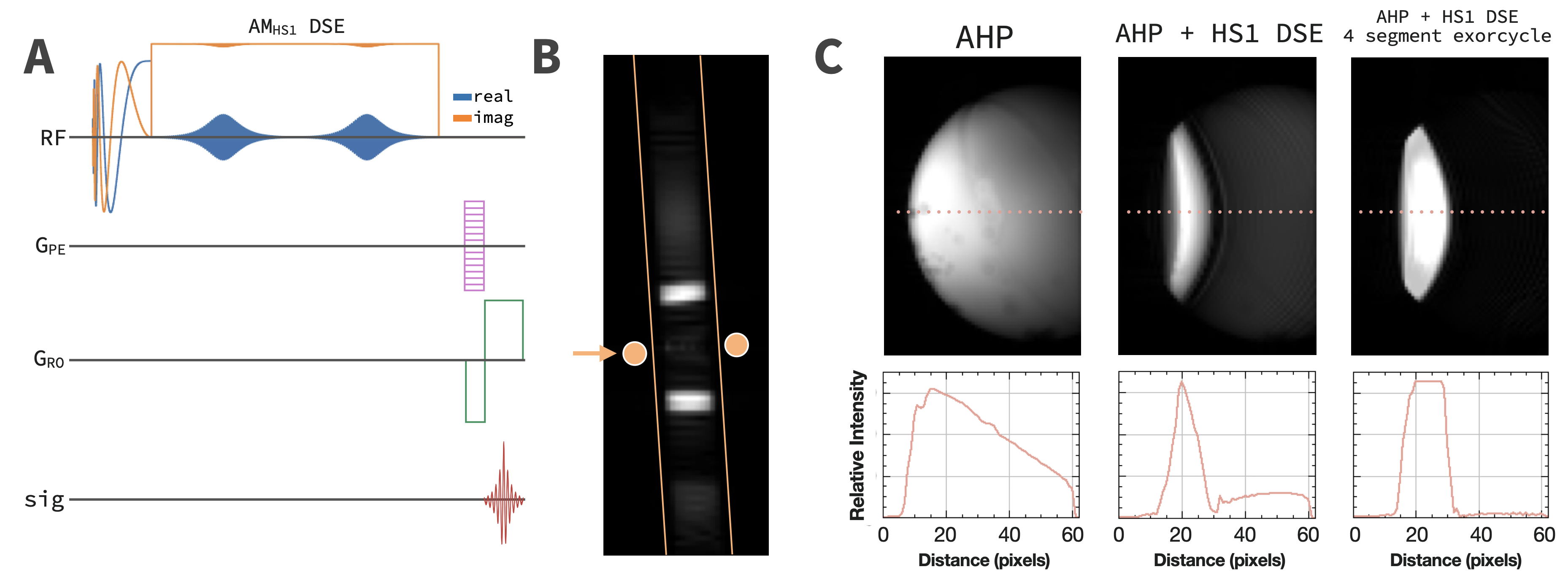
***

***Figure S3.*** *Experimental results of slice selection using an AM_HS1_ pulse and a surface coil on phantoms.* ***A)*** *Pulse sequence for AM_HS1_ applied as a double spin echo. Phase encode (G_PH_) and readout (G_RO_) gradients are used purely for slice visualization.* ***B)*** *Slice selection with a surface coil (arrow, cross-section) placed at the center of a tube with slices on either end.* ***C)*** *Slice selection with a surface coil placed on the left side of the sphere shown. (Left) Image after 4-ms AHP. (Middle) Image after 4-ms AHP + two consecutive AM_HS1_. (Right) Image after 4-ms AHP + two consecutive AM_HS1_ using four-step EXORCYCLE. Relative image intensity along dotted red line is shown below each image*.

**SAR and B_1_+ Modeling**

While AM_HS1_ may be applied at a variety of field strengths, low- to mid-field MRI scanners are likely the most practical for human scanning. To facilitate progress in the translation of an ultra-compact 0.7T scanner^[20]^, the modeling was designed to investigate the feasibility of achieving the desired $B_{1}^{+}$ strength and the corresponding SAR at 30.4 MHz (0.7T). Simulations of SAR and $B_{1}^{+}$ were performed using CST Studio (Darmstadt, Germany). The coil consisted of two loops formed from 4 mm wide copper traces, each measuring 24x30 cm. The corners were chamfered to reduce high electric current concentration. The two loops were separated by a 4 mm gap to provide sufficient self-capacitance. The loops were curved to a radius of 15 cm to fit the existing head coil setup within the 0.7T system. The RF coil was tuned and matched using lumped elements to 50 Ohms at 30.4 MHz and positioned ~4 cm from the human head. A 2-mm resolution anatomical voxel human model of Duke (34 y, male) from IT’IS^[21]^ was used to study the $B_{1}^{+}$ field and SAR effects induced by the coil. The coil was driven with 150 W of RF power and 20% duty cycle, via a proper 50 Ohm RF port.

The curved single coil with two loops shown (**Figure S4A**) produces an average $B_{1}^{+}$ of approximately 23.8 uT (Rabi frequency = 1013 Hz) in the head. Notably, in this ${\gamma B}_{1}^{+}$ gradient, a Rabi frequency of 1000 Hz occurs approximately halfway into the head, making it possible to perform slice selection deep in the brain (**Figure S4B**). The peak 10g SAR is 12.7 W/kg and the average SAR over the head is 3.07 W/kg. The average head SAR is below the limits for volume RF transmit coils under the normal operating mode. However, the peak 10g SAR falls under the first level controlled operating mode (under 6 min) according to IEC 60601-2-33:2022 guidelines^[22][23]^. Moreover, the local SAR limits over any 10 second period do not exceed 20 W/kg and 40 W/kg for normal operating mode and first level controlled operating mode, respectively.

***
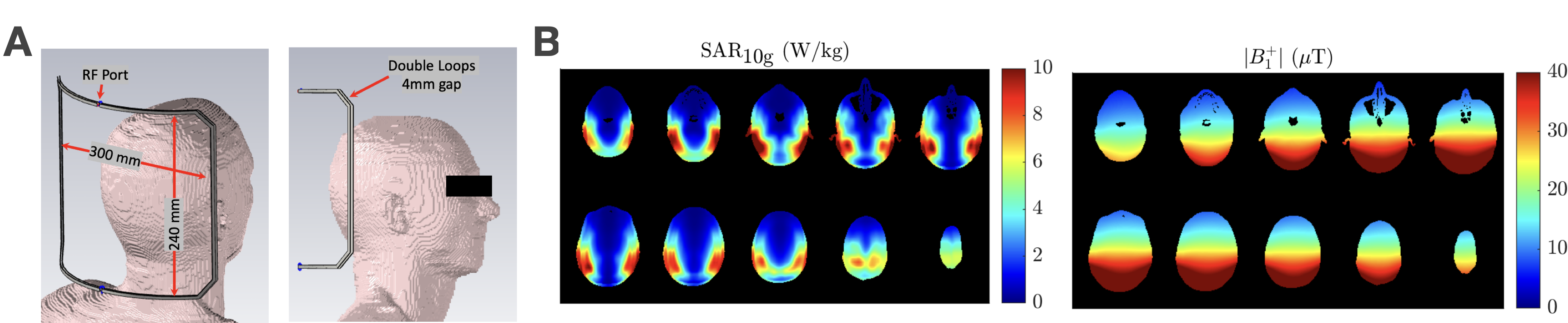
***

***Figure S4.*** *CST Studio 3D full-wave EM simulations of SAR and* $B_{1}^{+}$ *at 0.7T.* ***A)*** *Positioning of double-loop coil in the simulation setup with dimensions and RF input port labeled. The coil was driven with 150 W incident RF power and a 20% duty cycle.* ***B)*** *Simulation results of (Left) 10g SAR and (Right) |*$B_{1}^{+}$*| through slices of the head.*

***Correction of Low Flip Angle Slice Profile with Phase Modulation Shape Function***

To provide extra clarification on the method of slice profile correction for AM_HS1_ on a single coil at low flip angles using RF scaling (as in **Figure 7**), we show the effect of the extra minor correction using the shape function of the phase modulation. Initially there is a dip at the center of the slice profile which is more apparent at higher $\omega_{1y}^{'}\left( r_{\mathrm{center}} \right)$ values. Applying the phase modulation reduces this variation slightly **(Figure S5)**. Again, this is a minor correction, and the major emphasis of this correction should be placed on the FM shape function scaling. Simulation parameters were: $\omega_{1y}^{'}\left( r_{\mathrm{center}} \right)/2\pi$ = 1000 and 5000 Hz, $\omega_{1x}^{\max}/2\pi$ = 200 Hz, *T*_p_ = 11.98 ms (including the refocusing lobes), *R* = 10.4, M_0_ = [0 0 1], and an applied gradient $\omega_{G}^{'}/2\pi$ = 0 - 10000 Hz.


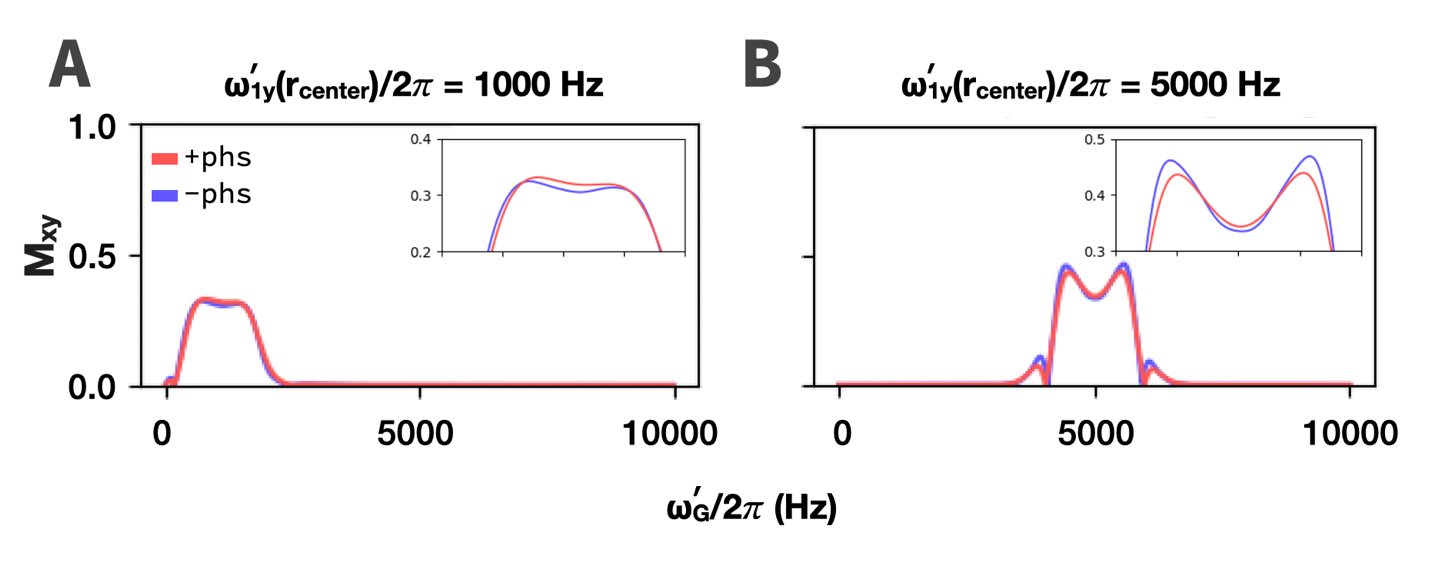


**Figure S5.** *Comparison of low flip angle slice profile for AM_HS1_ using a single coil with (+phs) and without (-phs) phase modulation shape function scaling for* ***A)*** $\omega_{1y}^{'}\left( r_{center} \right)/2\pi$ *= 1000 Hz and* ***B)*** $\omega_{1y}^{'}\left( r_{center} \right)/2\pi$ *= 5000 Hz. Both of these excitations use the FM shape function scaling.*
